# Atlantic West *Ophiothrix* spp. in the scope of integrative taxonomy: Confirming the existence of *Ophiothrix trindadensis* Tommasi, 1970

**DOI:** 10.1371/journal.pone.0210331

**Published:** 2019-01-23

**Authors:** Renata Aparecida dos Santos Alitto, Antonia Cecília Zacagnini Amaral, Letícia Dias de Oliveira, Helena Serrano, Karin Regina Seger, Pablo Damian Borges Guilherme, Maikon Di Domenico, Ana Beardsley Christensen, Luciana Bolsoni Lourenço, Marcos Tavares, Michela Borges

**Affiliations:** 1 Departamento de Biologia Animal, Universidade Estadual de Campinas, Campinas, São Paulo, Brasil; 2 Museu de Zoologia “Adão José Cardoso”, Universidade Estadual de Campinas, Campinas, São Paulo, Brasil; 3 Departamento de Biologia Celular, Universidade Estadual de Campinas, Campinas, São Paulo, Brasil; 4 Laboratório de Biologia Marinha, Universidade Estadual do Paraná, Paranaguá, Paraná, Brasil; 5 Centro de Estudos do Mar, Universidade Federal do Paraná, Pontal do Paraná, Paraná, Brasil; 6 Department of Biology, Lamar University, Beaumont, Texas, United States of America; 7 Museu de Zoologia da Universidade de São Paulo, São Paulo, Brasil; Laboratoire de Biologie du Développement de Villefranche-sur-Mer, FRANCE

## Abstract

We re-describe and confirm the validity of *Ophiothrix trindadensis* Tommasi, 1970 (Echinodermata: Ophiuroidea). This is a native species from Brazil, however it lacked a type series deposited in scientific collections. The recognition of *O*. *trindadensis* was made possible using integrative taxonomy applied to many specimens from the type locality (Trindade Island) as well as from different locations along the Brazilian coast (Araçá Bay and Estuarine Complex of Paranaguá). Initially, 835 specimens were studied and divided into four candidate species (CS) inferred from external morphological characters. Afterwards, the CSs were compared using integrative taxonomy based on external morphology, arm microstructures morphology (arm ossicle), morphometry, and molecular studies (fragments of the mitochondrial genes 16S and COI). Analyses indicated CS1 and CS2 as *O*. *trindadensis*, and CS3 as *O*. *angulata*, both valid species. CS4 remains *O*. cf. *angulata* as more data, including their ecology and physiology, are needed to be definitively clarified. Our integrative investigation using specimens from the type locality overcame the lack of type specimens and increased the reliable identification of *O*. *trindadensis* and *O*. *angulata*.

## Introduction

The Family Ophiotrichidae is one of the most interesting among Ophiuroidea, due to the brilliant colors of some species and their association with corals and sponges. This family has been revised multiple times, however, it continues to be problematic owing to the intraspecific variability [1, 2]. Currently, Ophiotrichidae has 169 species distributed across 16 genera [3], with ten species and two genera reported from Brazil [4].

The genus *Ophiothrix* Müller & Troschel, 1840 is considered taxonomically difficult due to the high degree of variability in morphological characters. Therefore, molecular characters have been used as a tool to detect and distinguish species that are morphologically similar [5–7]. A successful example applied to ophiotrichids is a study conducted across the Atlantic-Mediterranean area, which demonstrated the existence of three genetic and morphologically distinct lineages of *Ophiothrix*, although they have yet to be formally described [8, 9].

A total of 93 *Ophiothrix* species are recognized at present [3], and six are recorded from Brazil [4]: *O*. *ailsae* Tommasi, 1970; *O*. *angulata* (Say, 1825), *O*. *brachyactis* H. L. Clark, 1915, *O*. *rathbuni* Ludwig, 1882, *O*. *suensoni* Lütken, 1856, and *O*. *trindadensis* Tommasi, 1970. Of these, three are endemic: *O*. *ailsae* described from the coastal island Vitoria, State of São Paulo, *O*. *trindadensis* described from the remote oceanic island Trindade [10] and *O*. *rathbuni*, for which there is no type locality information [11]. Our attempts to locate the type series of the endemic Brazilian species *O*. *ailsae*, *O*. *trindadensis* and *O*. *rathbuni* at Oceanographic Institute of University of São Paulo, Museum of Zoology of University of São Paulo and Museum of Zoology of University of Campinas have been in vain.

The present study used an integrative approach to investigate *Ophiothrix* specimens and to clarify their taxonomic status and distribution in Brazil. External morphology was initially employed to propose candidate species. Then, the species boundaries among populations were evaluated using four independent character sets related to: i) external morphology, ii) arm microstructures morphology (arm ossicles), iii) morphometry, and iv) molecular data (nucleotide sequences of the mitochondrial genes 16S and COI).

One species of particular interest, *Ophiothrix trindadensis*, is redescribed here in detail. Since its type specimens were lost, we propose a neotype designation based on topotype specimen from Trindade Island following Article 75 of the International Code for Zoological Nomenclature [12].

## Material and methods

### 1) Study site and data collection

Brittle stars were collected from three sites in Brazil: i) Trindade (type locality of *Ophiothrix trindadensis*) and Martin Vaz Oceanic Archipelago, ii) Araçá Bay, and iii) Estuarine Complex of Paranaguá. These samples were then compared to those from three additional sites: i) São Pedro and São Paulo Archipelago–Brazil (molecular data), ii) Port Aransas, Texas, United States (morphological and molecular data), and iii) South Carolina, United States (type locality of *O*. *angulata*, morphological data) (S1 Fig). The geographic coordinates, substrate type, salinity, and main references for each locality sampled are shown in S1 Table. All specimens collected in the present study were fixed and preserved in 70% or 90% ethanol and deposited in the Museum of Zoology of the University of Campinas (ZUEC) labeled as ZUEC OPH (identification number) and Museum of Zoology of University of São Paulo labeled as MZUSP (identification number). The following is a brief description of each location as well as the sampling strategies that were used.

Trindade and Martin Vaz Oceanic Archipelago, Brazil (TMV) TMV is a group of one large (Trindade) and several smaller islands (Martin Vaz) located in the southwestern Atlantic approximately 1200 km east of Vitória, the capital of the Brazilian State of Espírito Santo. Trindade Island and the much smaller Martin Vaz Islands are only 49 km apart from each other. Trindade Island is 13.5 km^2^ in area and is almost totally composed of volcanic and subvolcanic rocks formed between the end of the Pliocene and the Holocene [13, 14].

During the project ProTrindade/CNPq, five trips to the TMV were conducted between 2012 and 2015. Most of the material reported was collected from scuba diving operations between 4–30 m, which resulted in 605 lots of shallow-water echinoderms. More information can be found in Anker et al. [15].

Araçá Bay, Brazil (AB) AB is located on the northeastern coast of the State of São Paulo, Brazil. It encompasses three beaches, two shores islets, three main stands of mangrove, rocky shores, and an extensive muddy sand flat extending to the subtidal zone. The latter comprises a vast intertidal flat up to 300 meters wide [16, 17].

Brittle stars were sampled during the project BIOTA/FAPESP–Araçá conducted between 2012 and 2016 from rubble bottom with a dredge and by hand associated with the sponge *Amphimedon viridis*.

Estuarine Complex of Paranaguá, Brazil (ECP) ECP is located on the southern coast of the State of Paraná, Brazil. The estuary measures 612 km^2^ and it is part of the largest remaining sectors of the Atlantic Rain Forest along the Brazilian coast. ECP is part of a large interconnected subtropical estuarine system comprised of two main water bodies, extensive sandy beaches, and rocky shores [18].

Brittle stars were sampled in 2014 in the intertidal zone of Banana Island and Perigo tidal flat by hand. All the specimens were associated with the sponge *Mycale* (*Zygomycale*).

São Pedro and São Paulo Archipelago, Brazil (SPSPA) SPSPA lies on the mid-Atlantic ridge, some 1000 km away from the coast of the State of Rio Grande do Norte, northeastern Brazil, and approximately 1960 km from the African coast [19]. SPSPA, the only Brazilian oceanic islands above the equator, is comprised of four larger islets plus several minor rocks, rising 4000 m from the ocean depths. The emerged area of the archipelago covers approximately 13000 m^2^ and is 420 m across at the greatest width [19, 20].

Two brittle stars identified as *Ophiothrix angulata* by Barboza et al. [21] were sampled by scuba diving in 2013. Logistics were supported by the PROARQUIPÉLAGO Program. The specimens were found associated with *Chaetopterus* polychaete tubes [21].

Port Aransas South Jetty, Texas, United States (TX-US) The South Jetty is at the north end of Mustang Island. The jetty, constructed in the early 1900's of large granite blocks quarried from Marble Falls, Texas, extends approximately 1 mile from the northern tip of Mustang Island into the Gulf of Mexico, stabilizing the entrance of the Corpus Christi Ship Channel [22, 23].

Brittle stars were collected between 2011 and 2012 on South Jetty by hand. The specimens were found in colonies of the sandy lobed tunicate *Eudistoma carolinense*.

South Carolina, United States (SC-US) A total of 18 specimens were borrowed from Smithsonian United States National Museum (USNM). Voucher E24138 contained nine specimens (dried) collected from South Creek, near the entrance in N. Edisto River, South Carolina in May 31, 1966. Voucher E28587 (in alcohol) with five specimens was sampled off South Carolina in September 12, 1980. Voucher 33710 (in alcohol) with four specimens was sampled in Calibogue Sound, SC in January 16, 1891.

### 2) Delimitation of the candidate species (CS) inferred from morphological characters

**Fig 1:**
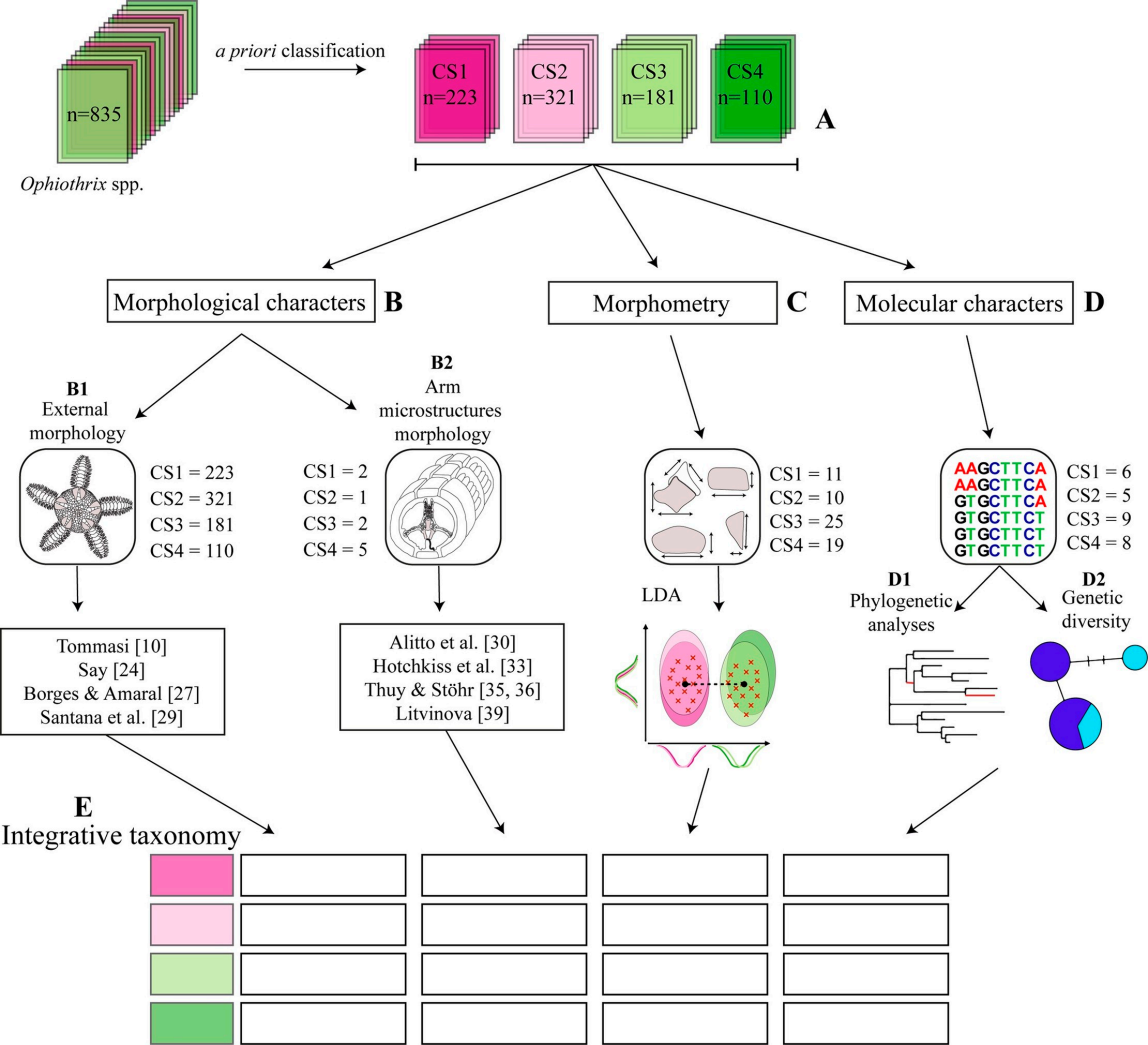
Schematic diagram of integrative taxonomy applied to *Ophiothrix* spp. (A) *A priori* classification inferred from morphological characters. (B) Morphological characters (B1) external morphology and (B2) arm microstructures morphology. (C) Morphometry: measurements and linear discriminant analysis (LDA). (D) Molecular characters: (D1) phylogenetic analyses (Maximum Parsimony, Bayesian Inference, and Maximum Likelihood) and (D2) genetic diversity and species delimitation test (genetic distances, AMOVA, haplotype network, bPTP). (E) Congruence framework of integrative taxonomy.

As a starting point for species validation, morphological hypotheses about the identity of CSs were proposed (step A, Fig 1A). They were based on characters usually reported as diagnostic in morphological overviews and keys [10, 24–30].

### 3) Morphological characters

#### External morphology

Comparisons of external characters of all CSs were made against descriptions and museum specimens of *Ophiothrix angulata* and *O*. *trindadensis*. In relation to *O*. *angulata*, the CSs were compared to its original description [24], redescription [29] and samples from type locality (South Carolina, United States) available from USNM (vouchers E24138–9 spms, E28587–5 spms, 33710–4 spms). They were also compared with the original description of *O*. *trindadensis* [10] (Step B1, Fig 1B).

#### Arm microstructures morphology

The best-preserved specimens of each CS were selected to study the arm ossicles, which were extracted from a small part of the proximal arm, between the fifth and the tenth segment. The arm segment was immersed in regular household bleach (NaClO) until the soft tissues were removed [31]. The ossicles were then washed with distilled water, air-dried, and prepared for examination with a scanning electron microscope (SEM) model JEOL JSM5800LV dx.doi.org/10.17504/protocols.io.tz6ep9e [PROTOCOL DOI].

The terms applied to the arm ossicles are based on literature [32–38] and are illustrated in Alitto et al. [30]. Arm vertebra joints were classified into types as proposed by Litvinova [39] (Step B2, Fig 1B).

### 4) Morphometry

Measurements were taken using an ocular micrometer and through the AxioVision VS program 40.4.8.20 (Carl Zeiss Microscopy, Germany) attached to a ZEISS Discovery V20 stereomicroscope for specimens less than 10 mm disc diameter and with a digital Mitutoyo CD-6 CS caliper for the larger specimens. A detailed list of measurements is presented in S2 Table and illustrated in S2 Fig.

The linear discriminant analysis (LDA) was applied using the R environment [40]. To avoid multicollinearity among morphological characters a correlation matrix was constructed and the variables that were significantly correlated were removed with a threshold value of 0.9. To investigate the differences in morphological characters, *lda* function (package MASS) [40, 41] was used to distinguish the four *Ophiothrix* CS. The classification rate of each CS was assessed by the “lda” function.

The predict function (package STATS) and table function (package BASE) were used to assess the classification based on the linear discriminants and to verify the model error. Visualization was performed using the package ggplot2 [42] (Step C, Fig 1C).

A permutational multivariate analysis of variance (PERMANOVA) [43] was conducted (function *adonis*) and homogeneity of group dispersions (functions *betadisper* and *permutest*) were then used to formally test whether morphological characters and dispersion differed between CSs (package vegan) [44] dx.doi.org/10.17504/protocols.io.tz5ep86 [PROTOCOL DOI].

*Ophiothrix angulata* specimens from the type locality (USNM) were predicted by the model to verify the CS to which they were most related.

### 5) Molecular characters

#### DNA extraction, amplification, and sequencing

DNA sequences used in phylogenetic analyses were obtained as follows. Samples of tube feet from the arms or gonads were soaked in two changes of Tris-EDTA (TE) buffer for 30 minutes each. The samples were then macerated in 500 μl of TE buffer with a pestle. A volume of 300 μl of a 10% Chelex 100 solution (BioRad) was added. The samples were then incubated at 55°C for 60 minutes, boiled for 8 minutes, cooled to room temperature, vortexed for 20 seconds followed by 60 seconds of centrifugation at 14,000 x*g*. The supernatant was decanted and kept refrigerated until use in polymerase chain reactions (PCR).

A fragment of the mitochondrial 16S gene was PCR-amplified using the forward primer 16S Sofi F (5′-CAGTACTCTGACTGTGCAA-3′) and the reverse primer 16S Sofi R (5′-GGAAACTATGATCCAACATC-3′) [8]. A fragment of the mitochondrial cytochrome oxidase subunit 1 (COI) gene was amplified using the forward primer COIf-L (5′-CCTGCAGGAGGGGGAGAYCC-3′) and the reverse primer COIa-H (5′-TGTATAGGCGTCTGGATAGTC-3′) [45].

PCR reactions were performed using PuReTaq Ready-To-Go™ PCR Beads for 25 μl reactions (GE Heathcare), complemented with 0.5 to 1 μl of 25mM MgCl_2_. Alternatively, PCR reactions were composed of 10 mM Tris-HCl, pH 8/1 mM KCl buffer, 1.5 mM MgCl_2_, and 200 μM each dNTP (Invitrogen). In both cases, 0.4 μM of each primer was used and template DNA concentration (ranging from 25.7 to 917.9 ng/μl) was tested.

Thermal cycling consisted of a single step at 94°C for 5 minutes, which was followed by 39 cycles (denaturation at 94°C for 45 seconds, annealing at 45.7°C to 51.3°C for 45 seconds, and extension at 72°C for 60 seconds) and a final extension at 72°C for 3 minutes on a thermal cycler (Eppendorf Mastercycler®).

PCR products were separated from excess primers and dNTP using a purification kit (Promega). Purified product was then used as a template for DNA sequencing reactions using BigDye Terminator (Applied Biosystems) and sequenced by *Serviço de Sequenciamento de DNA–SSDNA IQUSP* with the same primers used in the amplification reaction. A 372 bp fragment from 28 individuals was obtained for 16S and 466 bp fragment from 17 individuals for COI. All the sequences are deposited in GenBank (S3 Table). The nucleotide sequences were edited using BIOEDIT Sequence Alignment Editor v. 7.0.1 [46] dx.doi.org/10.17504/protocols.io.tz7ep9n [PROTOCOL DOI].

#### Phylogenetic analyses

All 16S and COI sequences were aligned with the MUSCLE algorithm [47], providing a unified matrix. All the sequences from *Ophiothrix angulata* (16S) and additional sequences from others Ophiotrichidae (16S, COI) available on GenBank were added to the matrix for comparison. *Amphipholis squamata* (Amphiuridae) was used as outgroup. All samples used are listed in S3 Table.

Three mitochondrial datasets were considered: i) 16S, ii) COI and iii) concatenated (COI+16S). Phylogenetic reconstructions were made on the basis of three different optimality criteria, Maximum Parsimony (MP), Bayesian Inference (BI), and Maximum Likelihood (ML) to assess whether there were any differences in the trees recovered with regard to the method used (Step D, Fig 1D).

The MP was performed with the TNT 1.5 program [48]. Most parsimonious trees were obtained by a heuristic search (best length was hit 100 times), using the new technology search option, which included sectorial searches, ratchet, tree drifting and tree fusing. The gaps were considered as fifth state. The cladograms had their nodes evaluated by the *Bootstrap* resampling test [49], based on 1000 pseudoreplicates using the *Traditional Search*.

For BI, the best-fitting model of molecular evolution was GTR+G, chosen based on the AIC and Hierarchical Likelihood Ratio Tests according to the estimation by MR.MODELTEST v.2.3 [50]. The BI analyses used one cold and three incrementally heated Monte Carlo Markov chains (MCMC) on two simultaneous runs. The standard deviation of the split frequencies between the two runs reached a value lower than c. 0.005 at two million generations, with one tree sampled every 100th generation, each using a random tree as a starting point and a temperature parameter value of 0.2 (the default in MrBayes). The first 25% of the total sampled trees were discarded as ‘burnin’ to achieve the MCMC log-likelihoods that had become stationary and converged. The analyses resulted in similar likelihood scores, with ESS > 200, as verified using TRACER.

The ML was performed with the MEGA v.7.0 program [51] using the Kimura 2-parameter model [52]. The statistical support was obtained with a bootstrap function using 1000 replicates.

The phylogenetic trees were visualized and edited in FIGTREE v.1.4.3 (http://tree.bio.ed.ac.uk/software/figtree/) and Adobe Illustrator dx.doi.org/10.17504/protocols.io.wimfcc6 [PROTOCOL DOI].

#### Analyses of the genetic diversity

The genetic distances between and within the different groups were estimated by *p-*distance using MEGA v. 6.0 [53], ignoring the alignment gaps in pairwise comparisons. Following Hart and Podolsky [6] and Pérez-Portela et al. [8], pairwise genetic *p*-distances >3% for 16S and >15% for COI were considered interspecific variations since most of the species already described have been differentiated by this minimum (Step D2, Fig 1D).

The concatenated matrix was submitted to the AMOVA (Analysis of Molecular Variance) test [54] to evaluate the genetic difference between and among the studied groups formed by the phylogenetic analyses. The overall fixation index (F_ST_) [55] was calculated to verify the gene flow between the groups. The test was simulated with 1000 permutations in Arlequin software v. 3.1 [56] (Step D2, Fig 1D).

The haplotype network was constructed based on the concatenated matrix by the median-joining network [57], implemented in Network software v. 5.0.0.1 (http://www.fluxus-engineering.com/) (Step D2, Fig 1D) 10.17504/protocols.io.t2peqdn [PROTOCOL DOI].

#### Species delimitation using bPTP

The phylogenetic tree generated by the Bayesian Inference was submitted to the Bayesian Poisson Tree Processes—bPTP [58] to test species boundaries. This test adds Bayesian support (BS) values to the nodes of the input tree and delimit species based on the Phylogenetic Species Concept (Step D2, Fig 1D).

The analyses were conducted on the web server (available at http://species.h-its.org/ptp/). The parameters for the run were 500000 MCMC generations, thinning of 100, and Burn-in of 0.25 dx.doi.org/10.17504/protocols.io.t2jeqcn [PROTOCOL DOI].

### 6) Integrative taxonomy

Species boundaries among populations of *Ophiothrix* were evaluated using four independent character sets in order to test: i) external morphology (Step B1, Fig 1B), ii) arm microstructures morphology (Step B2, Fig 1B), iii) morphometry (Step C, Fig 1C), and iv) molecular data (16S and COI fragments) (Step D, Fig 1D). The CSs were classified according to the congruence framework of Padial et al. [59] when there was concordance of, at least, three data sets analyzed (Step E, Fig 1E). Therefore, we highlight that molecular data was not weighted more heavily than morphological features dx.doi.org/10.17504/protocols.io.t2meqc6 [PROTOCOL DOI].

### 7) Ethics statements

Field work was authorized by the Chico Mendes Institute for Biodiversity Conservation–ICMBio, under the license number: 19887–1 to Cecília Amaral (AB) 38463–3 to Maristela Bueno (ECP) and 53376–1 to Marcos Tavares (TMV). Genetics data were registered at the National System for the Management of Genetic Heritage and Associated Traditional Knowledge–SisGen, according to Brazilian legislation Law number 13.123/2015 and Decree 8772/2016. Approval ID for this study was AC6C194.

## Results

### 1) Candidate Species (CS) inferred from morphological characters

**Fig 2:**
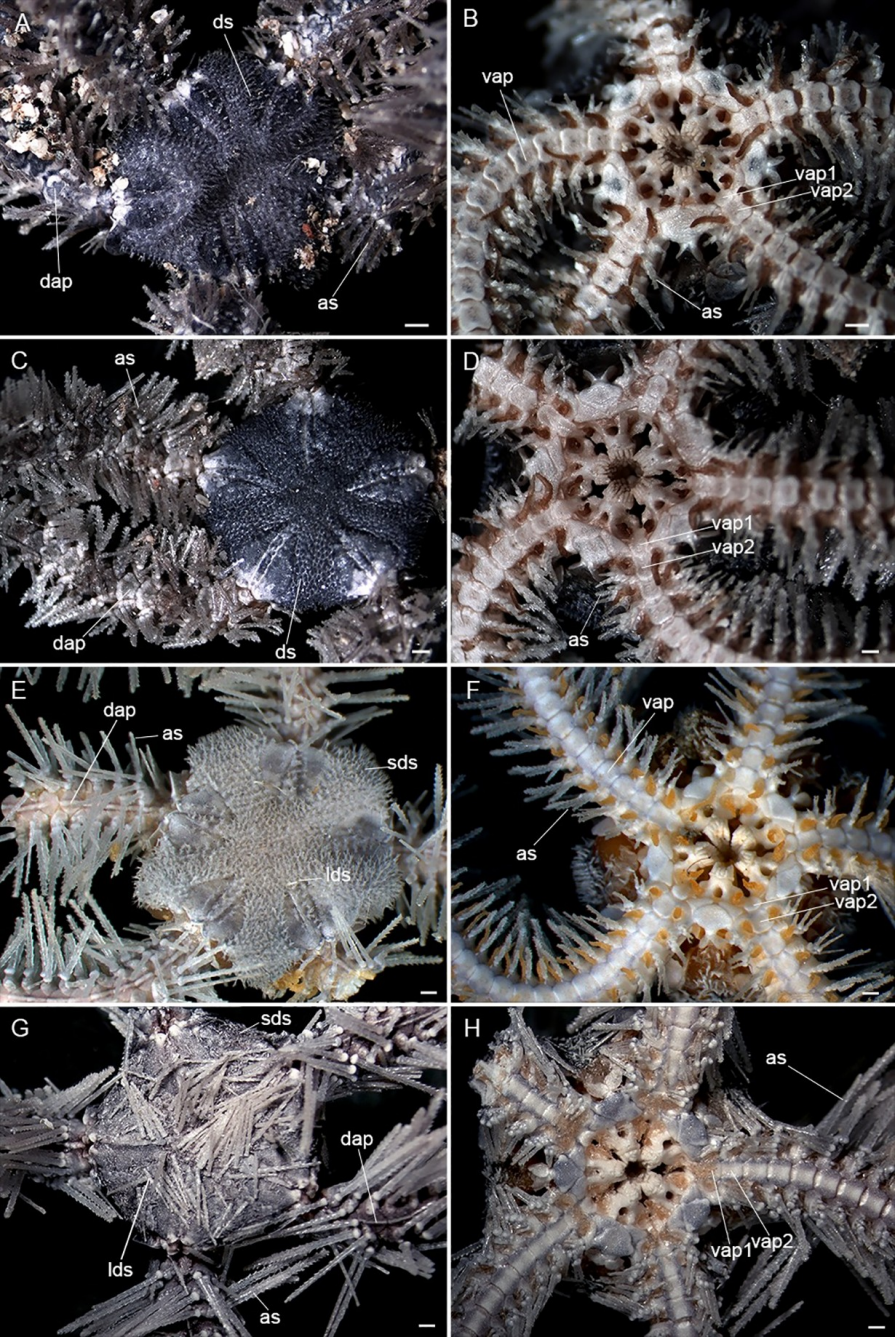
Candidate species of *Ophiothrix*. (A, B) CS1 –MZUSP 1426, Trindade Island. (C, D) CS2 –MZUSP 1425, Trindade Island. (E, F) CS3 –ZUEC OPH 2811, Araçá Bay. (G, H) CS4 –ZUEC OPH 2783, Estuarine Complex of Paranaguá. as: arm spine; dap: dorsal arm plate; ds: disc spine; lds: longer disc spine; sds: small disc spine; vap: ventral arm plate; vap1: first ventral arm plate; vap2: second ventral arm plate. Scale bars: 0.5 mm.

A total of 835 *Ophiothrix* specimens were analyzed and then classified into four CS: CS1 and CS2 from TMV and CS3 and CS4 from ECP and AB (Step A, Fig 1A). Table 1 and Fig 2 summarizes the main characteristics identified as diagnostic among the CS.

**Table 1:** Characters identified as diagnostic in the separation of *Ophiothrix* CSs.

Characters	*Ophiothrix* sp. CS1 Fig 2A and 2B	*Ophiothrix* sp. CS2 Fig 2C and 2D	*Ophiothrix* sp. CS3 Fig 2E and 2F	*Ophiothrix* sp. CS4 Fig 2G and 2H
Locality	TMV	TMV	ECP and AB	ECP and AB
Disc	Covered by hyaline spines up to 0.10 mm in length	Covered by hyaline spines up to 0.17 mm in length	Mostly covered by small hyaline spines of approximately 0.15 mm in length and some longer ones of approximately 0.8 mm in length scattered on the disc	Mostly covered by spines similar to those of the arms (denticulate) up to 2 mm in length and small hyaline spines of approximately 0.2 mm in length
Dorsal arm plates	With a prominent ridge at median portion similar to CS2 prominent ridge at median portion of the dorsal arm plates (dap). This structure was previously called “carena” (in Portuguese) by Tommasi	With a prominent ridge at median portion similar to CS1	Without a prominent ridge at median portion	Without a prominent ridge at median portion
Ventral arm plates	Distal edge concave, as long as wide, except the first which did not have a distal concavity	Distal edge concave, as long as wide, except the first and the second which did not have a distal concavity and are twice as long as wide (similar to a paddle shape)	Distal edge concave, slightly as long as wide	Distal edge concave, slightly as long as wide
Length of the longest arm spines	Two to three times greater than the width of one segment	Two to three times greater than the width of one segment	Two to three times greater than the width of one segment	More than three times larger than the width of one segment

AB, Araçá Bay, São Paulo, Brazil; ECP, Estuarine Complex of Paranaguá, Paraná, Brazil; TMV, Trindade and Martin Vaz Oceanic Archipelago, Brazil.

A detailed comparison of the CSs and six Brazilian *Ophiothrix* species listed by Barboza and Borges [4] were perfomed. The possibility that CSs belong to some of the species was rejected as follows: *O*. *rathbuni* has triangular and naked radial shields; *O*. *suensoni* possesses naked radial shields; *O*. *ailsae* has red bands on the arms and a disc covered only by trifid spines; and *O*. *brachyactis* possesses granules on dorsal disc.

The most similar species to CSs were *Ophiothrix angulata* and *O*. *trindadensis* as they have subpentagonal disc and the dorsal disc and radial shields are covered by bifid and/or trifid spines. However, some differences were noted among the specimens, particularly the length of the spines covering the dorsal disc, the dorsal and ventral arm plates, and number of arm spines.

### 2) Morphological characters

#### Taxonomic review

Three groups of *Ophiothrix* were formed according to their main morphological characters (Table 2). Group 1 included *O*. *trindadensis* from original description [10], CS1 and CS2. Their major characteristics are the absence of longer hyaline spines scattered on the disc, absence of denticulate spines on the disc, presence of a prominent ridge at median portion, termed carena by Tommasi, on dorsal arm plate, and five to 14 arm spines.

**Table 2:** Main morphological characters of all CSs compared with *Ophiothrix angulata* and *O*. *trindadensis*.

Characters	Group 1			Group 2				Group 3
	*O*. *trindadensis* Tommasi [10]	CS1	CS2	*O*. *angulata* Say [24]	*O*. *angulata* Santana et al. [29]	*O*. *angulata* Type locality USNM	CS3	CS4
Locality	TMV	TMV	TMV	SC-US	SC-US	SC-US	ECP, AB	ECP, AB
Disc (sub) pentagonal	✓	✓	✓	✓	✓	✓	✓	✓
Small hyaline spines and some longer ones scattered on the disc	X	X	X	ND	X	X	✓	X
Denticulate spines on the disc	X	X	X	ND	X	X	X	✓
Carena on dorsal arm plate	✓	✓	✓	ND	X	X	X	X
Ventral arm plates heart-shaped	✓	✓	✓	ND	✓	✓	✓	✓
Number of arm spines	9	5–13	7–14	7	5–9	5–7	5–8	4–9

Main morphological characters of all CSs with *Ophiothrix angulata*–original description [24], redescription [29] and specimens from type locality (USMN); *O*. *trindadensis*–original description [10].

AB, Araçá Bay, São Paulo, Brazil; ECP, Estuarine Complex of Paranaguá, Paraná, Brazil; ND, not described; SC-US, South Carolina, United States; TMV, Trindade and Martin Vaz Oceanic Archipelago, Brazil; USNM, Smithsonian Museum of Natural History; **✓** present; **X** absent.

Group 2 included all the *O*. *angulata* from original description [24], the redescription [29], samples from type locality (USNM) and CS3. Their main characteristics are the absence of longer hyaline spines scattered on the disc, absence of denticulate spines on the disc, absence of the carena on dorsal arm plate, and five to nine arm spines.

Group 3 included the CS4. Despite the similarities between *Ophiothrix angulata* and CS4, they were kept separate due to the denticulate disc spines on the latter.

#### Arm ossicles

A total of 10 specimens were analyzed: a) CS1 = 2 (samples MZUSP 1426, ZUEC OPH 2883); b) CS2 = 1 (MZUSP 1425); c) CS3 = 2 (MZUSP 1695, ZUEC OPH 2811); and d) CS4 = 5 (ZUEC OPH 2803, ZUEC OPH 2798, ZUEC OPH 2783, ZUEC OPH 2455, ZUEC OPH 2149). The dorsal, ventral, and lateral arm plates (S3 Fig) and arm vertebrae (S4 Fig) were examined.

The primary differences between the CSs are listed in S4 Table. All the differences are related to the dorsal and lateral arm plates. The dorsal arm plates of CS1 and CS2 were one and a half times as wide as long, distal region three to four times wider than the proximal one, with a carena at the median portion. The dorsal arm plates of CS3 and CS4 were as wide as long, distal and proximal regions at the same size, without a carena at median position. CS1 and CS2 have eight or more spine articulations on the lateral arm plates, while CS3 and CS4 have at most seven.

There were no differences in the vertebral ossicles. A true keel was observed in all CS. This was evident due the presence of a dorsal distal keel, dorsal projections, and depressions (large groove) connected by dorsal accessory muscles. These were also evident at the dorsal vertebral surface.

### 3) Morphometry

A total of 75 specimens were measured: CS1 = 11; CS2 = 10; CS3 = 25; CS4 = 19 and *Ophiothrix angulata* from type locality (USNM) = 10.

The LDA using all 17 morphological characters was effective in discriminating between the four *Ophiothrix* CSs (n = 65), supported by the results for within group dispersion BETADISPER (F_3,61_ = 7.1677, p<0.001), and PERMANOVA (F_3,61_ = 7.5725, p<0.001) analysis which confirmed significant multivariate differences in at least one of the CS.

**Fig 3:**
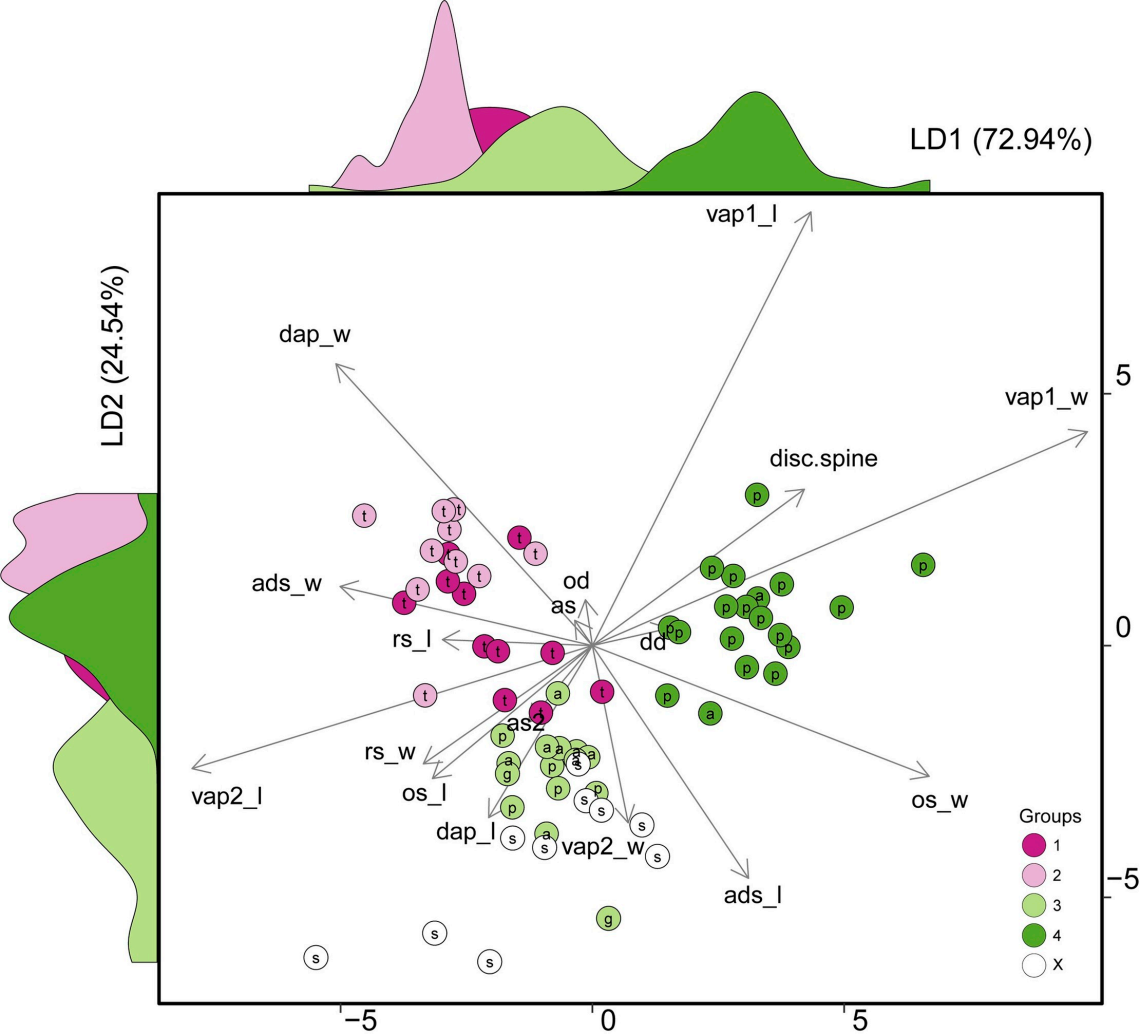
Axes 1 and 2 from linear discriminant analysis (LDA) based on 17 brittle stars’ characters. See S2 Table and S2 Fig for definitions of the morphological characters used for the morphometric analysis of *Ophiothrix* CSs. a: specimens from AB; g: specimen from TX-US; p: specimens from ECP; s: specimens from SC-US; t: specimens from TMV; X: *Ophiothrix angulata* from type locality (USNM).

The first and second linear discriminant axes described 72.94% and 24.54% of the among CSs variation in morphological characters, respectively (Fig 3). The classification success of LDA among the four CSs was 86.79%, showing CS3 and CS4 as the candidates classified correctly most often (100%), while CS1 was the most commonly misclassified (54%).

The width of the first ventral arm plate and width of the oral shield (vap1_w and os_w) were the morphological characters with highest positive coefficients in the first discriminant vector (LD1) indicating a strong contribution of these two characters in the multivariate separation model (Fig 3), separating mainly CS4 from the other CS. The highest negative coefficients were length of second ventral arm plate (vap2_l) and width of dorsal arm plate (dap_w), separating in two groups, one from CS1 and CS3 (TMV) and another from CS3 (AB, ECP) and *Ophiothrix angulata* from type locality.

Alternatively, the second discriminant vector (LD2) has length of first ventral arm plate (vap1_l) and width of dorsal arm plate (dap_w) as a positive coefficient and length of adoral shield (ads_l) and width of second ventral arm plate (vap2_w) as a negative coefficient, separating CS3 from the other CS.

### 4) Molecular characters

#### Phylogenetic analyses

**Fig 4:**
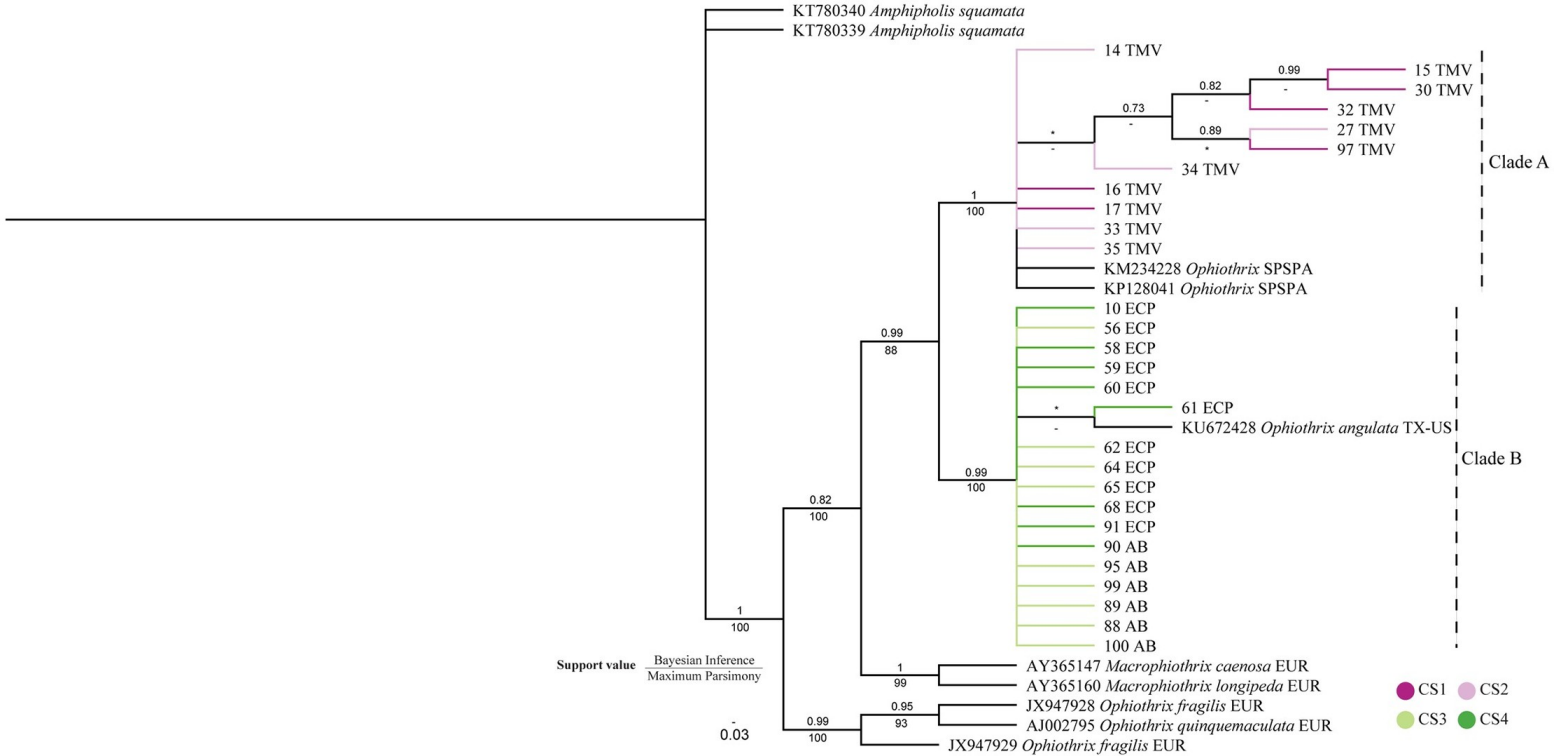
Cladogram inferred from the Bayesian analysis of 16S and COI sequences (concatenated). The numbers above the branches represent posterior probabilities. For the clades also inferred in the MP analysis, bootstrap values (%) are provided (below the branches). Branches are identified by individual codes (S3 Table) and their localities: AB: Araçá Bay, São Paulo, Brazil; ECP: Estuarine Complex of Paranaguá, Paraná, Brazil; EUR: Europe; SPSPA: São Pedro and São Paulo Archipelago, Brazil; TMV: Trindade and Martin Vaz Oceanic Archipelago; TX-US: Texas, United States. The scale bar represents the average nucleotide substitutions per site. Asterisks (*) indicate that the support value was lower than 0.7 (Bayesian Inference) or 70% (Maximum Parsimony), and a dash (-) indicates that the branch was not recovered in MP analysis.

The phylogenetic cladograms obtained either in the Bayesian analyses or in the Maximum Parsimony (concatenated matrix, Fig 4; 16S and COI individual matrices, S5 Fig) and Maximum Likelihood analyses (concatenated matrix, S6 Fig) of all datasets show two main clades with high support values, which were referred to as Clade A and Clade B. Clade A is formed by CS1, CS2 from TMZ and specimens from SPSPA, while Clade B is formed by CS3, CS4 from ECP and AB, and the single specimen from TX-US.

Five clades within Clade A were only found in Bayesian Inference, three of them with high support value (more than 0.8). Within Clade B, only one clade was found in Bayesian Inference and Maximum Parsimony, and two clades in Maximum Likelihood, but all with low support values.

#### Analyses of the genetic diversity

Genetic distances. The genetic distances observed in 16S and COI genes between CS1 and CS2 were low (1.4% for 16S, 3.5% for COI), as well as those observed between CS3 and CS4 (0.4% for 16S, 1.5% for COI). In contrast, high genetic distances were observed between specimens included in Clade A and those of Clade B which range from 11.4% to 12.1% for 16S gene and from 16.8% to 17.2% for COI gene (S5 Table).

AMOVA. AMOVA test revealed that 93.49% of total genetic variability occurred among the Clades A and B that were inferred in the phylogenetic analyses. Only 6.63% of total genetic diversity refers to variability within these groups. The overall fixation index (F_ST_) was extremely high (0.934) (S6 Table). These results indicate that the two groups are genetically distinct and well structured, indicating low or null gene flow between them.

**Fig 5:**
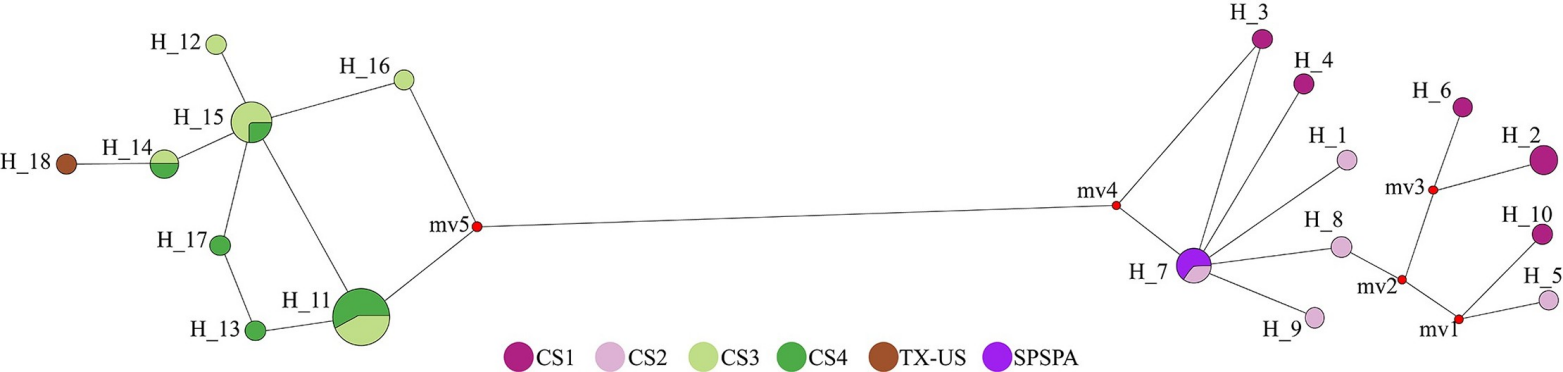
Median-joining haplotype network based on the concatenated matrix of 16S and COI gene sequences. Each circle represents one haplotype and their size is proportional to the number of individuals possessing it. SPSPA: Saint Peter and Saint Paul Archipelago, Brazil; TX-US: Texas, United States.

Haplotype Network. The haplotype network indicated the existence of two sharply divergent lineages (1: CS1+CS2 and 2: CS3+CS4+TX-US) lacking intermediate haplotypes. Divergence among clades was caused by 34 mutations. The first lineage (Fig 5, right) revealed a star-like pattern with H_7 as the ancestral haplotype surrounded by low-frequency (mostly private) haplotypes, separated by a few mutational steps. The second lineage (Fig 5, left) is composed of three dominant haplotypes (H_11, H_15 and H_14) surrounded by private haplotypes (Fig 5).

#### Species delimitation using bPTP

Clades A (CS1, CS2, SPSPA) and B (CS3, CS4, TX-US) found in the phylogenetic analyses were supported as distinct species by the PTP test. High values of posterior delimitation probabilities supported each of these clades (0.76 and 0.9 for Clade A and Clade B, respectively) (S7 Fig).

### 5) Integrative taxonomy

To integrate the results reported for the different operational criteria, a congruence framework was followed which considered that concordant divergence patterns among several taxonomic characters indicated that full lineage separation, as it was highly improbable that a coherent concordance pattern would emerge by chance [59].

**Fig 6:**
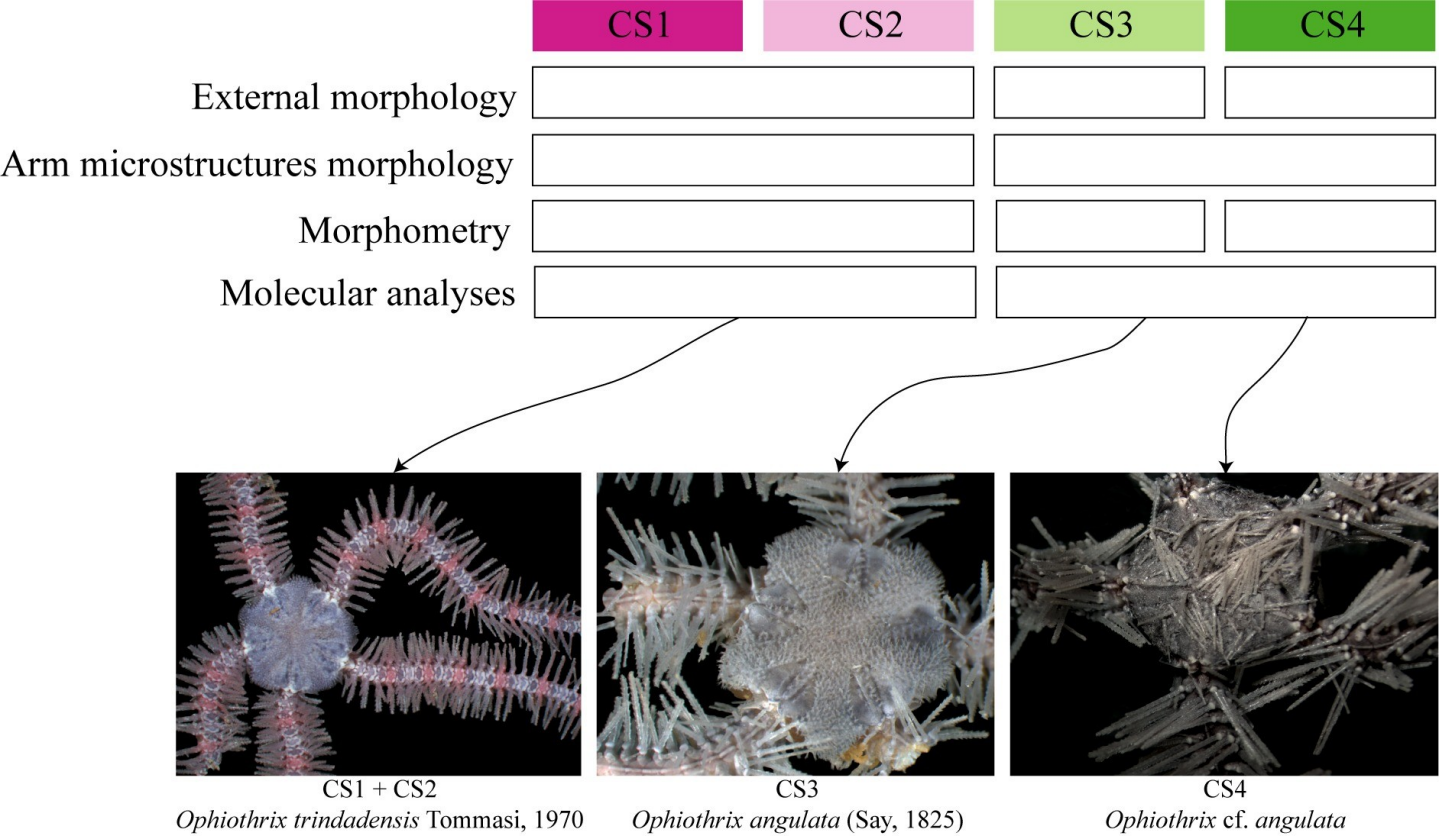
Synthetic representation of the integrative taxonomy approach within the *Ophiothrix* CSs. dap: dorsal arm plates; lap: lateral arm plates.

Concordance was found between the analyses based on external morphology, arm microstructures morphology (dorsal arm plates and lateral arm plates), morphometry, and molecular data (phylogenetic inferences, genetic distances, AMOVA, haplotype network, and bPTP) pointing to the existence of at least two species: (1) composed of CS1 and CS2 from TMV, and (2) composed of CS3 from ECP and AB. CS4 remains as *O*. cf. *angulata* as more data is needed to be definitively clarified (Fig 6).

In the following, we present the redescription of *Ophiothrix trindadensis* Tommasi, 1970, which is represented by CS1 and CS2, and *Ophiothrix angulata* (Say, 1825) as CS3. We propose CS4 as *O*. cf. *angulata* due to its different morphology, particularly the presence of spines on the disc similar to those of the arms (denticulate). The redescription of *O*. *trindadensis* contains the amended diagnosis, the description of an adult specimen (5.8 mm of dd) and juvenile specimen (less than 4 mm of dd) due to the importance of considering both in taxonomic practice [60–62]. We also added the variation in adult specimens, comparisons with *O*. *angulata*, taxonomic comments, remarks, and distribution.

### 6) Morphological description

***Ophiothrix trindadensis* Tommasi, 1970**

**CS1 and CS2**

**Neotype** MZUSP 1425 (sample 14).

**Type locality.** Trindade Island.

**Maximum size.** dd up to 8.5 mm (present study)

**Material examined.** 541 specimens. See S7 Table.

**Fig 7:**
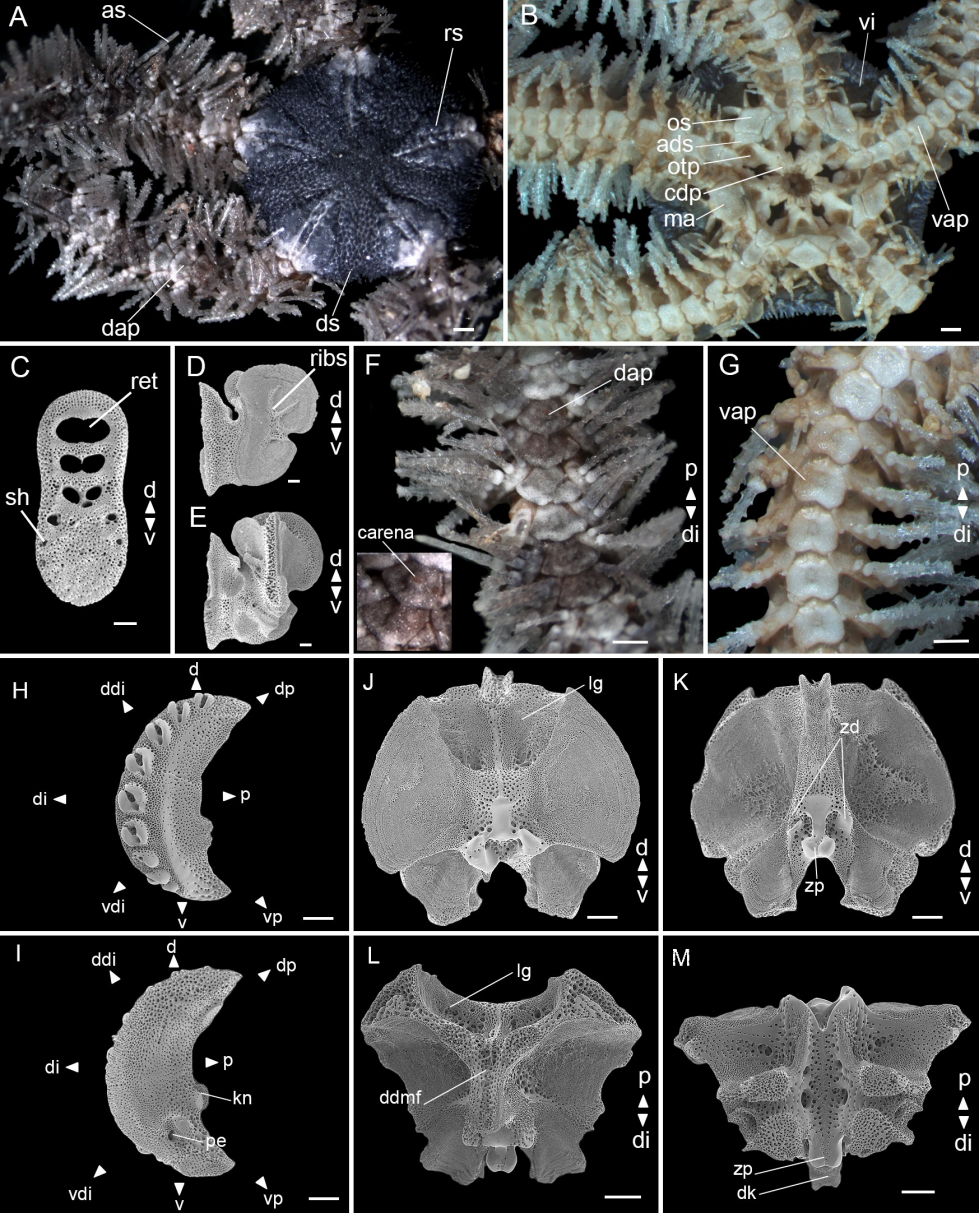
*Ophiothrix trindadensis* sample MZUSP 1425 (5.8 mm dd). (A) Dorsal view. (B) Ventral view. (C) Dental plate. (D) Abradial view of oral plate. (E) Adradial view of oral plate. (F) Detail of dorsal arm. (G) Detail of ventral arm. (H) Lateral arm plate–external side. (I) Lateral arm plate–internal side. (J-M) Vertebrae ossicle: (J) proximal surface. (K) distal surface. (L) dorsal surface. (M) ventral surface. ads: adoral shields; as: arm spine; cdp: cluster of dental papillae; dap: dorsal arm plate; d: dorsal; ddi: dorso-distal; ddmf: dorso-distal muscular fossae; di: distal; dk: distal keel; ds: disc spine; dp: dorso-proximal; kn: knob; lg: large groove; ma: madreporite; os: oral shields; otp: oral tentacle pore; p: proximal; pe: perforation; ribs: rib-like branching structures; ret: regular teeth; rs: radial shields; sh: small hole; v: ventral; vap: ventral arm plate; vdi: ventro-distal; vi: ventral interradius; vp: ventro-proximal; zd: zygocondyle; zp: zygosphene. Stereomicroscope photos (A, B, F, G), scale bar equal to 0.5 mm. SEM photos (C-E, H-M), scale bar equal to 100 μm.

**Amended diagnosis**. Pentagonal disc covered by small hyaline spines. Dorsal arm plates fan-triangular, distal region three to four times greater than the proximal, and with a prominent carena at median portion. Eight to eleven long arm spines (longest about three arm joints), vitreous and denticulate (Fig 7).

**Description of the adult neotype.** Disc (dd: 5.8 mm). Pentagonal, covered by small hyaline spines. Radial shields triangular, three times as long as wide, one-third of dd, united distally and separated proximally (Fig 7A). Ventral interradius covered by scales with small spines, the same as the dorsal (Fig 7B).

Mouth plating. Oral shields almost twice as wide as long, tapered proximally and with a slight projection at the distal edge. Madreporite larger than other oral shields, but with a similar shape. Adoral shields broadened distally and united proximally. Depression between two oral plates. A cluster of dental papillae on the apex of the jaw. Infradental papillae and lateral oral papillae absent. Oral tentacle pore visible (Fig 7B). Dental plate with equal width all over, an outer column of small holes at each edge on ventral half, dorsal half with fenestrations and a septum (Fig 7C). Abradial view of oral plate with rib-like branching structures on muscle attachment area (Fig 7D). Adradial view of oral plate with a large, dorsal, spoon-shaped depression on muscle attachment area (Fig 7E).

Arms. Dorsal arm plates fan-triangular, one and a half times as wide as long, and with a prominent carena at median portion (Fig 7F). Ventral arm plates as long as wide, with a slightly notch at distal edge, proximal edge with a small tip (Fig 7G). One tentacle scale. Eight to eleven long arm spines (longest about three arm joints), vitreous and denticulate, the second to ventral-most being the smallest and the ventral-most modified into a hook with hyaline teeth facing proximal side (Fig 7G).

Lateral arm plates (Fig 7H and 7I): general outline: arched (wrapped around the arm); without constriction; projecting ventro-proximalwards; ventro-distal tip not projecting ventralwards. Outer surface ornamentation: trabecular intersections protruding to form knobs approximately the same size as stereom pores. Outer proximal edge: surface lined by discernible band of different stereom structure, restricted to central part; without spurs; central part protruding; surface without horizontal striation. Arm spine articulations: nine, on elevated portion not bordered proximally by ridge; directly adjacent to the distal edge; arranged over entire distal edge; middle spine articulation(s) larger; distance between spine articulations equidistant. Lobes merged at their proximal tips by smooth connection; lobes parallel, equal-sized, bent, with tilted orientation; stereom with perforations; sigmoidal fold absent. Inner side: dominated by two separate central knobs with a ridge; without additional dorsal structure; single large perforation.

Vertebrae: zygospondylous of universal type and keeled. Large groove on proximal side of vertebrae dorsally corresponding to distalwards projecting dorso-distal muscular fossae of distal side (Fig 7J). Zygocondyles dorsalwards converging and zygosphene fused with pair of zygocondyles (Fig 7K). Narrow dorsal keel protruding distalwards far beyond vertebra edge, matching large dorsal groove proximally (Fig 7L and 7M).

**Variation in the adult specimens**. Disc covering: with larger and/or smaller spines. Shape of oral shield: with straight and/or rounded edges. Adoral shields: separated or united proximally. Ventralmost arm spine modified into a hook with hyaline teeth facing the disc: often starting at 8^th^ or 9^th^ segment but occasionally from the 5^th^ segment. Spine on the first dorsal arm plate: sometimes present and easily observable. Number of arm spines: eight to eleven, but one was identified with 14.

**Color patterns in preserved adult specimens.** Most common are (S8 Fig): purple disc, gray and purple arms; purple disc, pink and purple arms; purple with white–disc and arms. Some others are: red disc and arms; orange disc and purple arms; and brown with white–disc and arms.

**Juvenile specimens—less than 4 mm of dd**

**Material examined.** 4 specimens. See S7 Table.

**Fig 8:**
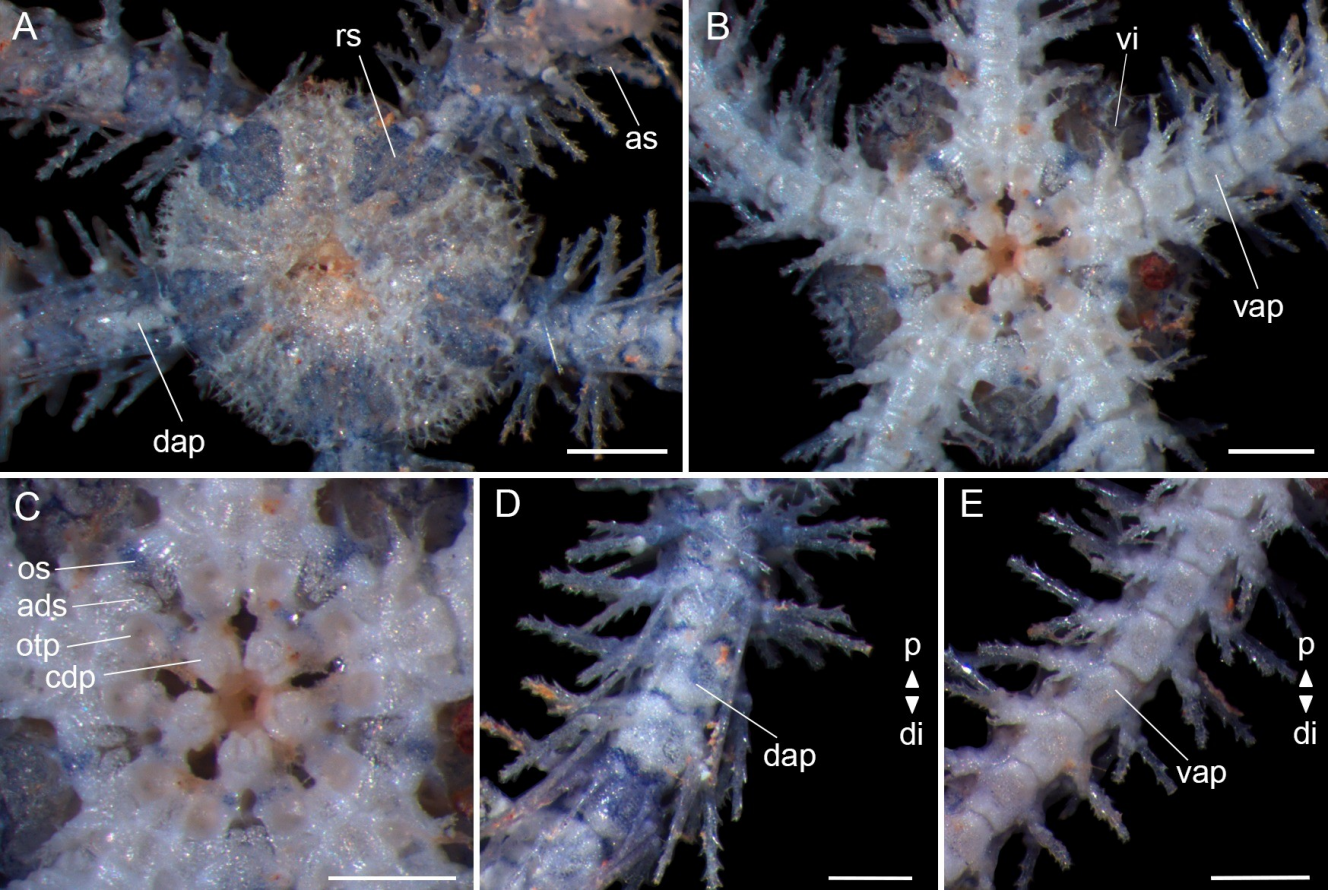
*Ophiothrix trindadensis*–juvenile with 2.1 mm of dd (MZUSP 1687). (A) Dorsal view. (B) Ventral view. (C) Detail of the oral view. (D) Detail of dorsal arm. (E) Detail of ventral arm. ads: adoral shields; as: arm spine; cdp: cluster of dental papillae; dap: dorsal arm plate; di: distal; os: oral shields; otp: oral tentacle pore; p: proximal; rs: radial shields; vap: ventral arm plate; vi: ventral interradius. Stereomicroscope photos, scale bar equal to 0.5 mm.

Disc (dd = 2.1 mm): subpentagonal, covered by small hyaline spines. Radial shields triangular, two times as long as wide, one-fourth of dd, united distally and separated proximally (Fig 8A). Ventral interradius covered by scales with small spines, the same as the dorsal (Fig 8B). Oral shields almost three times as wide as long, tapered proximally and without a slight projection at the distal edge. Madreporite not distinguishable. Adoral shields broadened distally and united proximally. Depression between two oral plates. A cluster of dental papillae on the apex of the jaw and without lateral oral papilla. Oral tentacle pore visible (Fig 8C).

Arms. Dorsal arm plates fan-triangular, one and a half times as wide as long, and with a prominent carena at median portion only in specimens greater than 2.9 mm of dd (Fig 8D). Ventral arm plates twice as long as wide, and the notch at distal edge is smaller than the notch in adult specimens (Fig 8E). One tentacle scale. Four to eight long arm spines (longest about three arm joints), vitreous and denticulate, the second to ventral-most being the smallest and the ventral-most modified into a hook with hyaline teeth facing the disc (Fig 8E).

**Color patterns in preserved juvenile specimens.** Most common are purple radial shields; purple and white on disc and arms; and with a bright whitish circle in the center of the disc.

**Taxonomic comments.** The main differences between *O*. *trindadensis* and *O*. *angulata* are: i) prominent carena at median portion of the dorsal arm plates of *O*. *trindadensis*, which is absent in *O*. *angulata*; and ii) eight to eleven long arm spines in *O*. *trindadensis*, while *O*. *angulata* is frequently characterized by at most seven arm spines. Barboza et al. [21] described the dorsal arm plates of the *Ophiothrix angulata* from SPSPA with the distal edge slightly lobed, which we assume to be the carena found on *O*. *trindadensis*. The specimens from SPSPA were deposited in the collection of the National Museum of Rio de Janeiro, Brazil, but unfortunately, they were not accessible. In addition to the carena on dorsal arm plates, the low genetic differences between specimens from TMV and SPSPA shows the existence of just one species in TMV and SPSPA, *O*. *trindadensis*.

**Remarks.** *Ophiothrix trindadensis* was collected from rubble bottom or associated with algae *Lithothamnion*. It has also been sampled in corals [10].

**Distribution**. Tropical Atlantic (realm), Tropical Southwestern Atlantic (province): São Pedro and São Paulo Islands [21], Northeastern Brazil [63], Eastern Brazil [64], Trindade and Martin Vaz Islands [10].

The present study samples occurred at depths ranging from 7 to 25 m.

***Ophiothrix angulata* (Say, 1825)**

**Type locality.** Charleston Harbour, South Carolina (United States).

**CS3.** Araçá Bay and Estuarine Complex of Paranaguá, Brazil; South Carolina and Texas, United States.

**Maximum size.** dd up to 10 mm [27].

**Material examined.** 181 specimens. See S7 Table.

**Amended diagnosis**. Pentagonal disc, covered by small and hyaline spines and some longer ones scattered on the disc. Dorsal arm plates fan-triangular, as wide as long. Five to eight long arm spines (longest about three arm joints), vitreous and denticulate.

**Description.** Original description in Say [24], neotype description in Santana et al. [29], microstructures description in Alitto et al. [30].

**Taxonomic comments.** Specimens from type-locality (USNM, voucher E24138) were compared with our samples. The only difference observed was the shape of the ventral arm plates: slightly concave distally in our specimens while it was cordiform in specimens from type-locality. Two characters were not observed in the specimens from USNM and the present study: i) carena on dorsal arm plates and ii) disc covered by spines similar to those of the arms (denticulate).

**Remarks.** In Araçá Bay, it was collected from rubble bottom with a dredge or associated with the sponge *Amphimedon viridis*. In Estuarine Complex of Paranaguá, it was sampled with the sponge *Mycale* (*Zygomycale*).

**Distribution.** See Alitto et al. [30]. Specimens from type locality occurred at depths ranging from 13 to 66 m. The present study samples occurred at depths ranging from intertidal to 20 m.

***Ophiothrix* cf. *angulata***

**CS4.** Araçá Bay and Estuarine Complex of Paranaguá, Brazil.

**Maximum size.** dd up to 7.8 mm (present study).

**Material examined.** 110 specimens. See S7 Table.

**Amended diagnosis**. Pentagonal disc, covered by small and hyaline spines, mostly covered by spines similar to those of the arms (denticulate). Dorsal arm plates fan-triangular, as wide as long. Five to eight long arm spines (longest about three arm joints), vitreous and denticulate.

**Fig 9:**
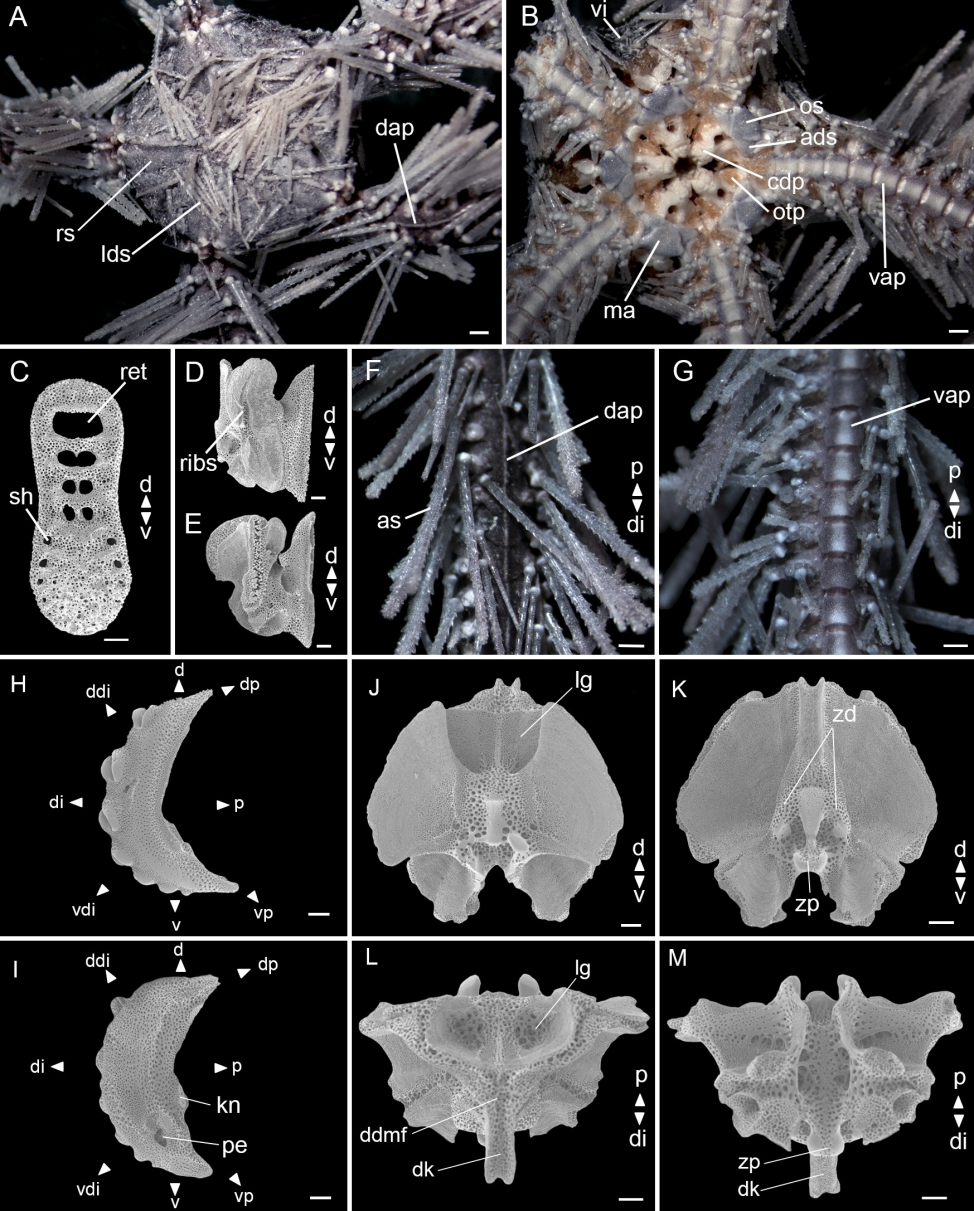
*Ophiothrix* cf. *angulata* ZUEC OPH 2783 (6.4 mm dd). (A) Dorsal view. (B) Ventral view. (C) Dental plate. (D) Oral plate—abradial view. (E) Oral plate—adradial view. (F) Detail of dorsal arm. (G) Detail of ventral arm. (H) Lateral arm plate–external side. (I) Lateral arm plate–internal side. (J-M) Vertebrae ossicle: (J) proximal surface. (K) distal surface. (L) dorsal surface. (M) ventral surface. ads: adoral shields; as: arm spine; cdp: cluster of dental papillae; d: dorsal; dap: dorsal arm plate; ddi: dorso-distal; di: distal; dk: distal keel; ddmf: dorso-distal muscular fossae; dp: dorso-proximal; kn: knob; lds: longer disc spine; lg: large groove; ma: madreporite; os: oral shields; otp: oral tentacle pore; p: proximal; pe: perforation; ribs: rib-like branching structures; ret: regular teeth; rs: radial shields; sh: small holes; v: ventral; vap: ventral arm plate; vdi: ventro-distal; vi: ventral interradius; vp: ventro-proximal; zd: zygocondyle; zp: zygosphene. Stereomicroscope photos (A, B, F, G), scale bar equal to 0.5 mm. SEM photos (C-E, H-M), scale bar equal to 100 μm.

**Description of the adult.** Disc (dd: 6.4 mm) pentagonal, covered by small hyaline, bifid and/or trifid spines and some longer ones scattered on the disc. Radial shields triangular, twice as long as wide, one-third of dd, united distally and separated proximally, covered by bifid and/or trifid spines (Fig 9A). Ventral interradius covered by scales, except near the oral shields and bursal slits (Fig 9B).

Mouth plating. Oral shields as long as wide, tapered proximally and with a slight projection at the distal edge. Madreporite larger than other oral shields, but with a similar shape. Adoral shields broadened distally and separated proximally. Depression between two oral plates. A cluster of dental papillae on the dental plate. Infradental papillae and lateral oral papillae absent. Oral tentacle pore visible (Fig 9B). Dental plate with equal width all over, an outer column of small holes at each edge on ventral half, dorsal half with fenestrations and a septum (Fig 9C). Abradial view of oral plate with rib-like branching structures on muscle attachment area (Fig 9D). Adradial view of oral plate with a large, dorsal, spoon-shaped depression on muscle attachment area (Fig 9E).

Arms: dorsal arm plates fan-triangular, as long as wide and contiguous (Fig 9F). Ventral arm plates straight or slightly concave on distal edge, proximal edge straight, lateral edges convex and slightly as long as wide (Fig 9G). One tentacle scale. Five to eight long arm spines (longest about four arm joints), vitreous and denticulate, the second to ventral-most being the smallest and the ventral-most modified into a hook with hyaline teeth facing the disc (Fig 9E).

Lateral arm plates (Fig 9H and 9I): general outline: arched (wrapped around the arm); without constriction; ventral portion projecting ventro-proximalwards; ventro-distal tip not projecting ventralwards. Outer surface ornamentation: trabecular intersections protruding to form knobs approximately the same size as stereom pores. Outer proximal edge: surface lined by discernible band of different stereom structure, restricted to central part; without spurs; central part protruding; surface without horizontal striation. Arm spine articulations: seven, on elevated portion not bordered proximally by ridge; directly adjacent to the distal edge; arranged over entire distal edge; all similar in size; distance between spine articulations dorsalwards increasing. Lobes merged at their proximal tips by smooth connection; lobes parallel, equal-sized, bent, with tilted orientation; stereom with perforations; sigmoidal fold absent. Inner side: dominated by two separate central knobs with a ridge; without additional dorsal structure; single large perforation.

Vertebrae: zygospondylous of universal type and keeled. Large groove on proximal side of vertebrae dorsally corresponding to distalwards projecting dorso-distal muscular fossae of distal side (Fig 9J). Zygocondyles dorsalwards converging and zygosphene fused with pair of zygocondyles (Fig 9K). Narrow dorsal keel protruding distalwards far beyond vertebra edge, matching large dorsal groove proximally (Fig 9L and 9M).

**Taxonomic comments.** A total of five specimens were considered juvenile (less than 4 mm of dd). Spines similar to those of the arms (denticulate) were observed on the disc even in the smallest samples. The number of arm spines of most adults was 6 to 7, however three specimens had 8 arm spines.

**Remarks.** In Araçá Bay, specimens were collected from rubble bottom with a dredge or associated with the sponge *Amphimedon viridis*. In the Estuarine Complex of Paranaguá, it was sampled with the sponge *Mycale* (*Zygomycale*).

**Distribution.** Tropical Atlantic (realm), Tropical Southwestern Atlantic (province): Southeastern Brazil (present study). The present study samples occurred at depths ranging from intertidal to 20 m.

## Discussion

The combined integrative taxonomy based on external morphology, arm microstructures morphology, morphometry, and molecular data has confirmed the identity of two species of *Ophiothrix*. The candidate species that were previously identified as CS1, CS2, CS3, and CS4 are now classified as: 1) *Ophiothrix trindadensis* Tommasi, 1970, formed by CS1 and CS2, and redescribed here in detail, 2) *Ophiothrix angulata* as CS3. The CS4 was described as *O*. cf. *angulata* as more data are needed to be definitively clarified.

The conclusion that CS1 and CS2 are *Ophiothrix trindadensis* is due to the four distinct analyses (external morphology, arm microstructures morphology, morphometry, and molecular) that distinguished it from CS3 and CS4, and because they are from the type locality of *O*. *trindadensis*, Trindade Island. As the analyses failed to separate CS1 from CS2, they have been combined as a single species with intraspecific morphological variation, such as larger and/or smaller spines on the disc.

The main character observed in the arm ossicles of *Ophiothrix trindadensis* was a prominent ridge at median portion of the dorsal arm plates (DAP). This structure was previously called “carena*”* (in Portuguese) by Tommasi [10] and is rediscribed here as a diagnosis. Another diagnostic feature related to DAP was that the distal region was three to four times wider than the proximal one, unlike *O*. *angulata*, which is the same width. And the last feature was the number of the spine articulations on the arm ossicles, and consequently, the arm spines on the lateral arm plates. *O*. *trindadensis* can have up to 14 arm spines, unlike *O*. *angulata*, which has at most nine.

The morphometric analysis of the *Ophiothrix trindadensis* yielded two characters that separated it from the others CS: i) length of the second ventral arm plate, and ii) width of dorsal arm plate. For these two characters, we made initial comments and predictions, which aided in the separation of the CSs before the tests. We did not know if these characters would be significant *a priori*. This demonstrates the power of morphometry in the separation of species for taxonomic studies. Measures of ventral and dorsal arm plates are highly indicative of separation of *Ophiothrix* species and should be considered in diagnosis.

The specimens from Trindade and Martin Vaz Archipelago (TMV) formed a clade, here named Clade A, with strong support in both phylogenetic analyses. The level of genetic divergence between Clades A and B was high (11.4–12% and 16.8–17.2% for 16S and COI, respectively). These levels of 16S and COI divergence are similar to genetic distances found between *Ophiothrix fragilis* and *O*. *quinquemaculata* (about 9.5% for 16S and 16.5% for COI) in Europe [8, 9].

The hypothesis that the TMV specimens were *Ophiothrix angulata* was discarded for three reasons. First because the original description of *O*. *angulata* [24] depicts seven arm spines. Second because the genetic divergence of 16S gene between TMV specimens and *O*. *angulata* (KU672428) was high (11.7–12.1%). And third due to the absence of the carena on dorsal arm plates in the *O*. *angulata* from type locality (USNM).

Another feature of interest was the similarity between the specimens from TMV and São Pedro and São Paulo Archipelago (SPSPA). The genetic divergence of 16S gene between these two localities was low (0.6–1.2%), which is indicative of intraspecific variations. According to Barboza et al. [21], the specimens from SPSPA were deposited in the collection of the National Museum of Brazil (NMRJ), but unfortunately, they were not found. Despite this, Barboza et al. [21] described the dorsal arm plates of the specimens with distal edge slightly lobed, which we interpret to be the carena present on *Ophiothrix trindadensis*.

The diagnosis that CS3 from Clade B is *Ophiothrix angulata* was made for three reasons. First is because of the similarities between CS3 with the original description of Say [24] and redescription of Santana et al. [29]. Second is due to the low genetic divergence of 16S gene between CS3 and *O*. *angulata* (KU672428) (0.8%). The sample KU672428 is from Port Aransas, Texas, USA, which is the nearest place of the *O*. *angulata* type locality (Charleston Harbour, South Carolina, USA). And finally, due to the morphological similarities between CS3 and *O*. *angulata* from type locality (USNM).

CS4 was redescribed here as *Ophiothrix* cf. *angulata*. This conclusion was due to the low genetic divergence (0.9% and 1.5% for 16S and COI, respectively) between CS4 and *Ophiothrix angulata* (KU672428) and the great similarity between CS4 and *O*. *angulata* from type locality (USNM). Despite this, we believe that it is necessary to continue the studies of CS4 for the following reasons: i) the morphometric analysis separated the CS4 from other CSs; ii) the presence of disc spines similar to those of the arms (long and denticulate); and iii) necessity of comparison with other *Ophiothrix* species.

CS3 was frequently found in Araçá Bay (AB), while CS4 was often in Estuarine Complex of Paranaguá (ECP). This must be addressed in future studies, particularly when considering the environments in which they live. The AB is a more saline region with oceanographic features distinct from ECP. Furthermore, the specimens were sampled in different sponges, CS3 in *Amphimedon viridis* while CS4 in *Mycale* (*Zygomycale*). This observation has prompted several questions concerning their ecology and physiology as well. What degree can the differences observed on disc coverage (presence or absence of denticulate spines) could be related to their i) biological substrate? ii) the different salinities and/or depths sampled? In any case, we emphasize the need for further studies, including ones concerning their ecological traits.

A true keel was observed in all CS. Unfortunately, knowledge about the vertebrae is still scarce, with only a few studies describing possible correlations between vertebral morphologies and ecological specialization [65–68]. The need for further studies such as behavioral observation and mechanical testing of individual arm segments is necessary to elucidate the function of these structures.

Our results demonstrate the utility of applying integrative taxonomic approaches for brittle stars specimens, particularly at the species level according to Padial et al. [59]. This method has demonstrated great efficiency and accuracy in the taxonomic delimitation of several groups, for instance beetles, as shown by Arribas et al. [69]. However, implementation of integrative taxonomy may be problematic for hidden species and/or higher taxonomic levels, such as family and genera. To assist in this issue, Korshunova et al. [70] developed several operational rules rooted in biological facts, which were successfully applied to a group of mollusks. These certainly may be applied for other metazoan groups.

We highlight the importance of depositing samples in museums, as they document historical and current patterns of biological diversity, which cannot be replaced. Additionally, future studies should consider the use of morphometry combined with scanning electron microscopy in order to detect patterns undetected by the naked eye.

## Supporting information

S1 Fig Study sites and data collection. Triangles represent the samples that were collected during the present study: Trindade and Martin Vaz Oceanic Archipelago, Araçá Bay, and the Estuarine Complex of Paranaguá. Circles represent the location of samples used in our comparisons: São Pedro and São Paulo Archipelago (molecular data), South Carolina (morphological data), and Texas (morphological and molecular data). The molecular data were obtained from GenBank. The shapefile was imported from the Natural Earth project (the 1:50m resolution version).
(TIF)

S2 Fig Illustrations of the 17 morphological characters used for the morphometric analysis. The abbreviations and definitions are described in S2 Table.
(TIF)

S3 Fig Arm ossicles of *Ophiothrix* CSs. dorsal (dap), ventral (vap), and lateral arm plates (lap). CS1 MZUSP 1426: (A) dap; (B) vap; (C,D) lap. CS2 MZUSP 1425: (E) dap; (F) vap; (G, H) lap. CS3 ZUEC OPH 2811: (I) dap; (J) vap; (K,L) lap. CS4 ZUEC OPH 2783: (M) dap; (N) vap; (O, P) lap. d: dorsal; ddi: dorso-distal; di: distal; dp: dorso-proximal; p: proximal; pe: perforation; v: ventral; vdi: ventro-distal; vp: ventro-proximal. Scale bars: 100 μm.
(TIF)

S4 Fig Four main sides of the vertebrae ossicle of *Ophiothrix*. Proximal view (pv), distal view (div), dorsal view (dov), ventral view (vv). CS1 MZUSP 1426: (A) pv; (B) div; (C) dov; (D) vv. CS2 MZUSP 1425: (E) pv; (F) div; (G) dov; (H) vv. CS3 ZUEC OPH 2811: (I) pv; (J) div; (K) dov; (L) vv. CS4 ZUEC OPH 2783: (M) pv; (N) div; (O) dov; (P) vv. d: dorsal; di: distal; dk: distal keel; lg: large groove; p: proximal; v: ventral. Scale bars: 100 μm.
(TIF)

S5 Fig Phylogenetic relationships inferred from the 16S (left) and COI (right) matrices by Bayesian analysis (MrBayes analysis). Numbers above and below branches represent the support values for BI (posterior probabilities) and MP (bootstrap values in %), respectively. The MP tree was not represented. Branches are identified by individual codes and their localities (AB: Araçá Bay, São Paulo, Brazil; ECP: Estuarine Complex of Paranaguá, Paraná, Brazil; EUR: Europe; SPSPA: São Pedro and São Paulo Archipelago, Brazil; TMV: Trindade and Martin Vaz Oceanic Archipelago). The scale bar represents the average nucleotide substitutions per site. Asterisks (*) indicates that the support value was lower than 70% (MP) or 0.7 (BI), and a dash (-) indicates that the branch was not recovered.
(TIF)

S6 Fig Cladogram inferred from the Maximum Likelihood of 16S and COI sequences (concatenated). The numbers in nodes represent the support values (bootstrap). Branches are identified by individual codes (S3 Table) and their localities: AB: Araçá Bay, São Paulo, Brazil; ECP: Estuarine Complex of Paranaguá, Paraná, Brazil; EUR: Europe; SPSPA: São Pedro and São Paulo Archipelago, Brazil; TMV: Trindade and Martin Vaz Oceanic Archipelago; TX-US: Texas, United States. The scale bar represents the average nucleotide substitutions per site. Asterisks (*) indicate that the support value was lower than 70%.
(TIF)

S7 Fig Species delimitation hypothesis obtained in the maximum likelihood solution of the Bayesian Poisson Tree Processes (bPTP) analysis. Branches in red connect haplotypes included in same species, in blue distinct species. Branches are identified by individual codes (S3 Table) and their localities: AB: Araçá Bay, São Paulo, Brazil; ECP: Estuarine Complex of Paranaguá, Paraná, Brazil; EUR: Europe; SPSPA: Saint Peter and Saint Paul Archipelago, Brazil; TMV: Trindade and Martin Vaz Oceanic Archipelago; TX-US: Texas, United States.
(TIF)

S8 Fig *Ophiothrix trindadensis*–color patterns in some preserved specimens. (A) ZUEC OPH 2907, dd = 5.8 mm. (B) MZUSP 1661, dd = 5.3 mm. (C) MZUSP 1687, dd = 3,1 mm. (D) MZUSP 1450, dd = 6.1 mm. (E) MZUSP 1686 dd = 4.2 mm. (F) ZUEC OPH 2883, dd = 8.5 mm. Stereomicroscope photos, scale bar equal to 1 mm.
(TIF)

S1 Table Study sites that brittle stars were collected (TMV, AB, ECP) and compared (SPSPA, TX-US, SC-US). AB, Araçá Bay; ECP, Estuarine Complex of Paranaguá; SC-US, South Carolina, United States; SPSPA, São Pedro and São Paulo Islands; TMV, Trindade and Martin Vaz Oceanic Archipelago; TX-US, Texas, United States; USNM, United States National Museum;–absent.
(DOCX)

S2 Table Abbreviations and definitions of the 17 morphological characters used for the morphometric analysis. (DOCX)

S3 Table Specimens used in the phylogenetic analyses with their locality, museums vouchers, and GenBank accession numbers for DNA sequences. AB, Araçá Bay, Brazil; AM, Australian Museum; ECP, Estuarine Complex of Paranaguá, Brazil; EUR, Europe; FMC, French Mediterranean Coast; IC, individual codes in the present study; NMRJ, National Museum of Rio de Janeiro, Brazil; ROS, Roscoff/France; SPSPA, São Pedro and São Paulo Archipelago; TMV, Trindade and Martin Vaz Oceanic Archipelago, Brazil; TX-US, Texas, United States; ZUEC OPH, Scientific Collection of Ophiuroidea in Museum of Zoology of the University of Campinas.
(DOCX)

S4 Table Comparative table of the differences in dorsal and lateral arm plates between the CS. (DOCX)

S5 Table Genetic distance (%) between and within the CS. Genetic distance between the CSs estimated from the mitochondrial gene fragments 16S (downward triangle) and COI (top triangle). In the gray diagonal line, average genetic distance (%) of both genes within the groups of sequences.
AB, Araçá Bay, São Paulo, Brazil; CS, Candidate Species inferred from morphological characters; ECP, Estuarine Complex of Paranaguá, Paraná, Brazil; EUR, Europe; Nc, Value not calculated because of the inexistence of groups’ sequences for the considered gene; SPSPA, Saint Peter and Saint Paul Archipelago, Brazil; TMV, Trindade and Martin Vaz Oceanic Archipelago; TX-US, Texas, United States. # value of genetic distance within the group not calculated because only one sequence was considered in the analysis.
(DOCX)

S6 Table Analysis of Molecular Variance (AMOVA) for the population structuring scenario given by the groups I and II. Group I is composed of the specimens from Trindade and Martin Vaz Oceanic Archipelago, Saint Peter and Saint Paul Archipelago–which corresponds to Clade A inferred in the phylogenetic analysis. Group II is composed of the specimens from Estuarine Complex of Paranaguá, Araçá Bay, and Texas–which corresponds to Clade B inferred in the phylogenetic analysis.
(DOCX)

S7 Table Material used in this study. MZUSP = Museum of Zoology of University of São Paulo. ZUEC OPH = Museum of Zoology of the University of Campinas. * = juvenile specimens (less than 4 mm of dd).
(XLSX)
