# Supplementary figures and images for: Atlantic West Ophiothrix spp. in the scope of integrative taxonomy: Confirming the existence of Ophiothrix trindadensis Tommasi, 1970
Source: PLoS One. 2019 Jan 23;14(1):e0210331. doi: 10.1371/journal.pone.0210331 (PMC6343879; doi:10.1371/journal.pone.0210331)

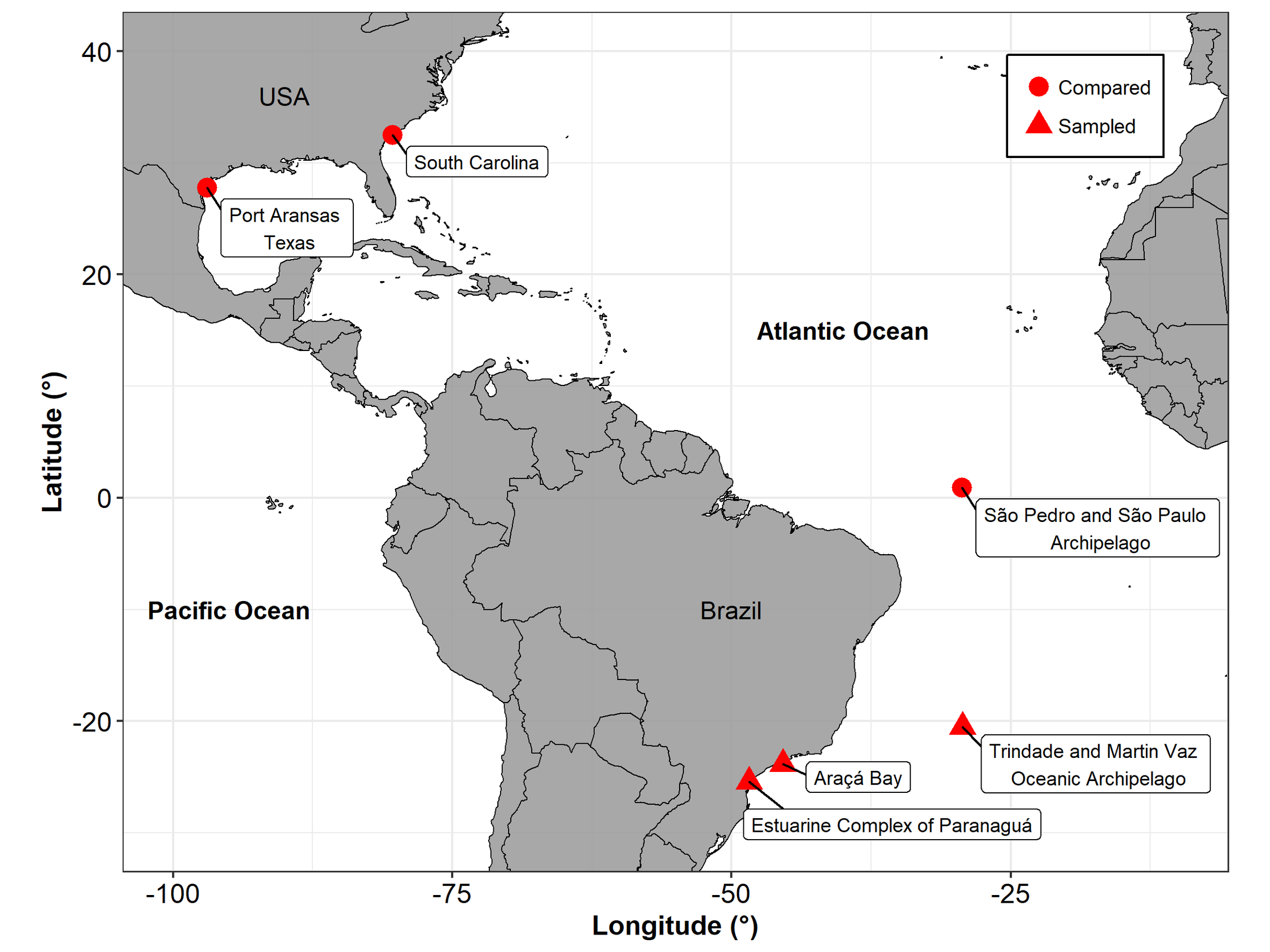

Supplement: S1 Fig — Triangles represent the samples that were collected during the present study: Trindade and Martin Vaz Oceanic Archipelago, Araçá Bay, and the Estuarine Complex of Paranaguá. Circles represent the location of samples used in our comparisons: São Pedro and São Paulo Archipelago (molecular data), South Carolina (morphological data), and Texas (morphological and molecular data). The molecular data were obtained from GenBank. The shapefile was imported from the Natural Earth project (the 1:50m resolution version). (TIF) [file pone.0210331.s001.tif]

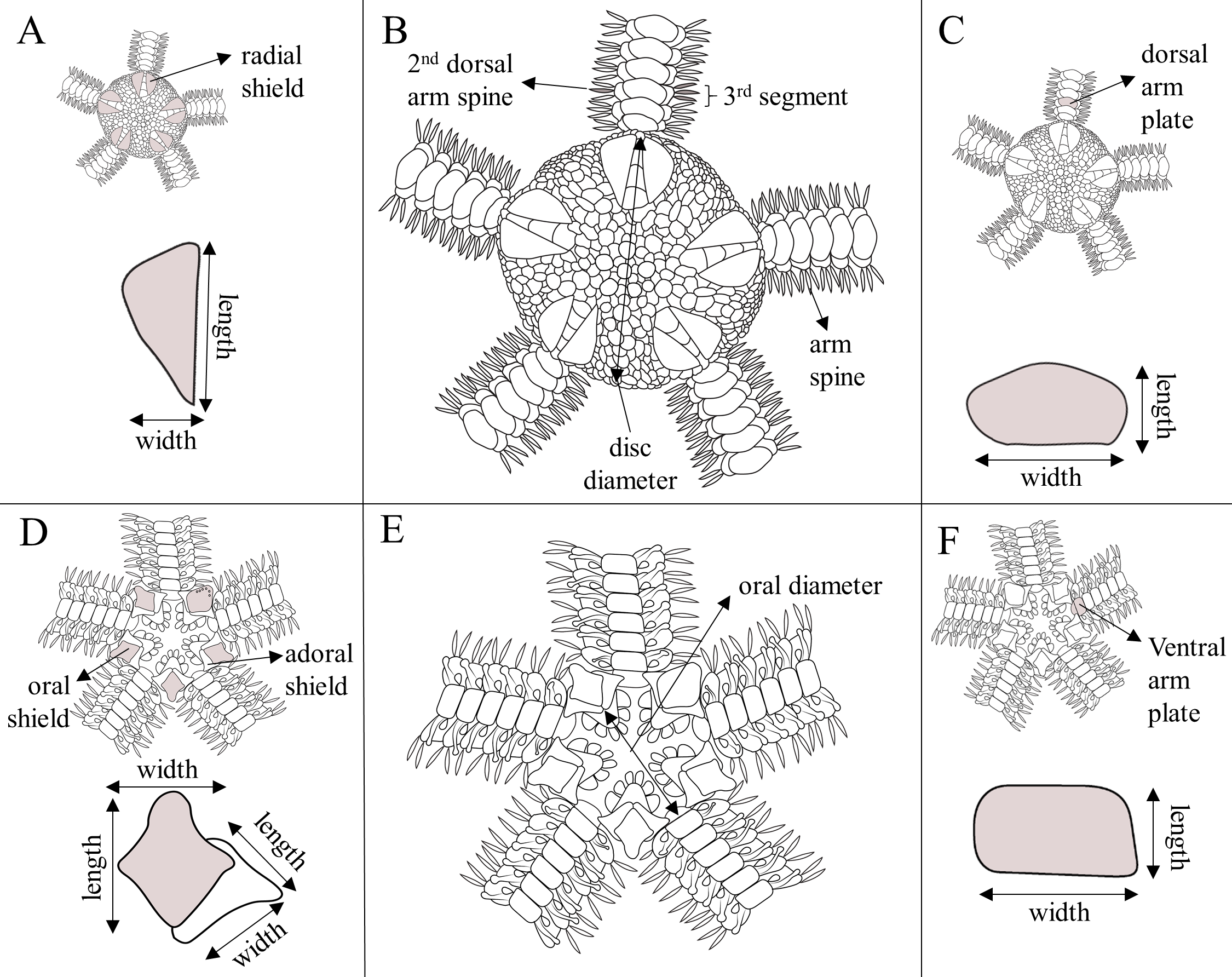

Supplement: S2 Fig — The abbreviations and definitions are described in S2 Table. (TIF) [file pone.0210331.s002.tif]

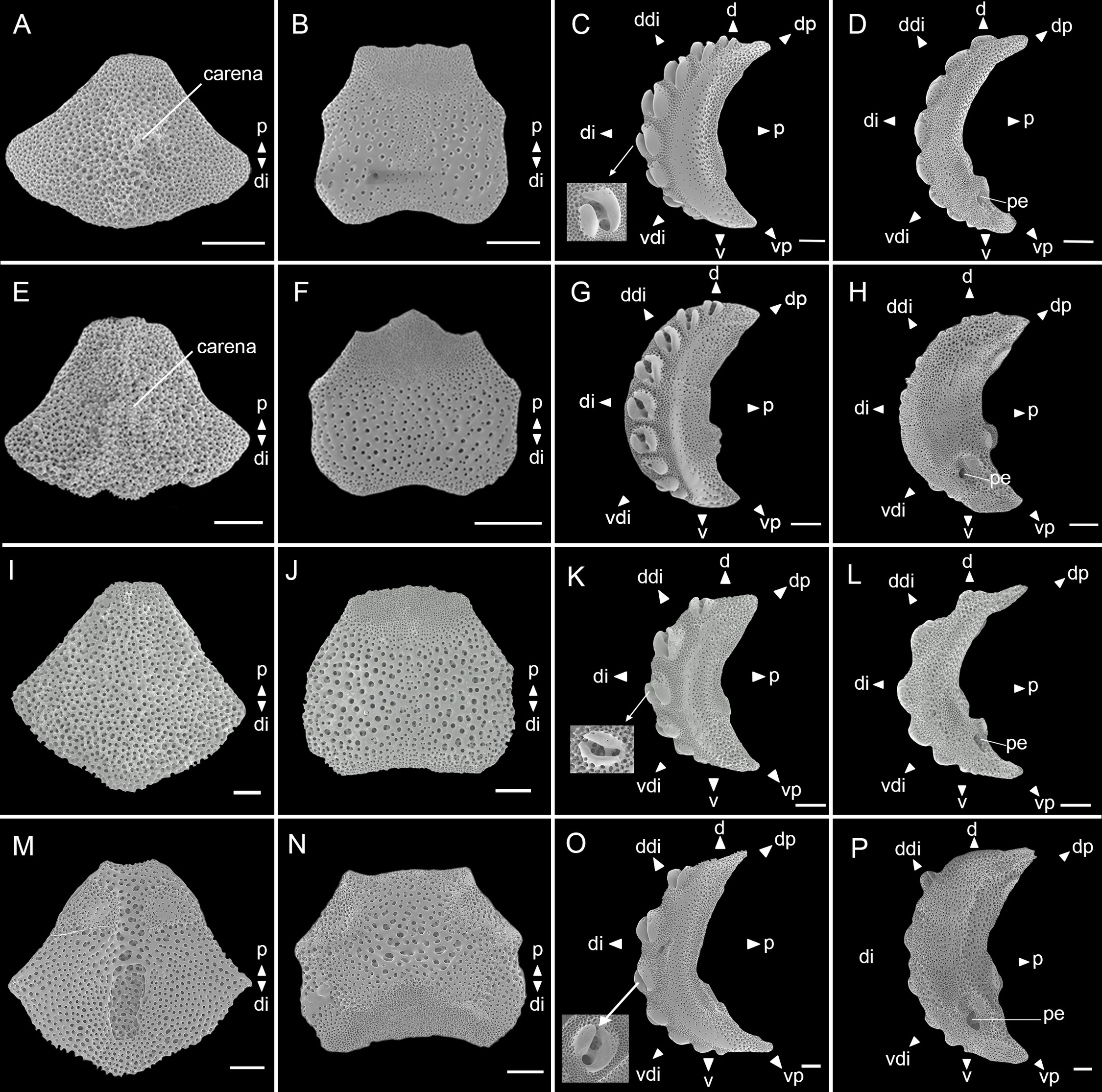

Supplement: S3 Fig — dorsal (dap), ventral (vap), and lateral arm plates (lap). CS1 MZUSP 1426: (A) dap; (B) vap; (C,D) lap. CS2 MZUSP 1425: (E) dap; (F) vap; (G, H) lap. CS3 ZUEC OPH 2811: (I) dap; (J) vap; (K,L) lap. CS4 ZUEC OPH 2783: (M) dap; (N) vap; (O, P) lap. d: dorsal; ddi: dorso-distal; di: distal; dp: dorso-proximal; p: proximal; pe: perforation; v: ventral; vdi: ventro-distal; vp: ventro-proximal. Scale bars: 100 μm. (TIF) [file pone.0210331.s003.tif]

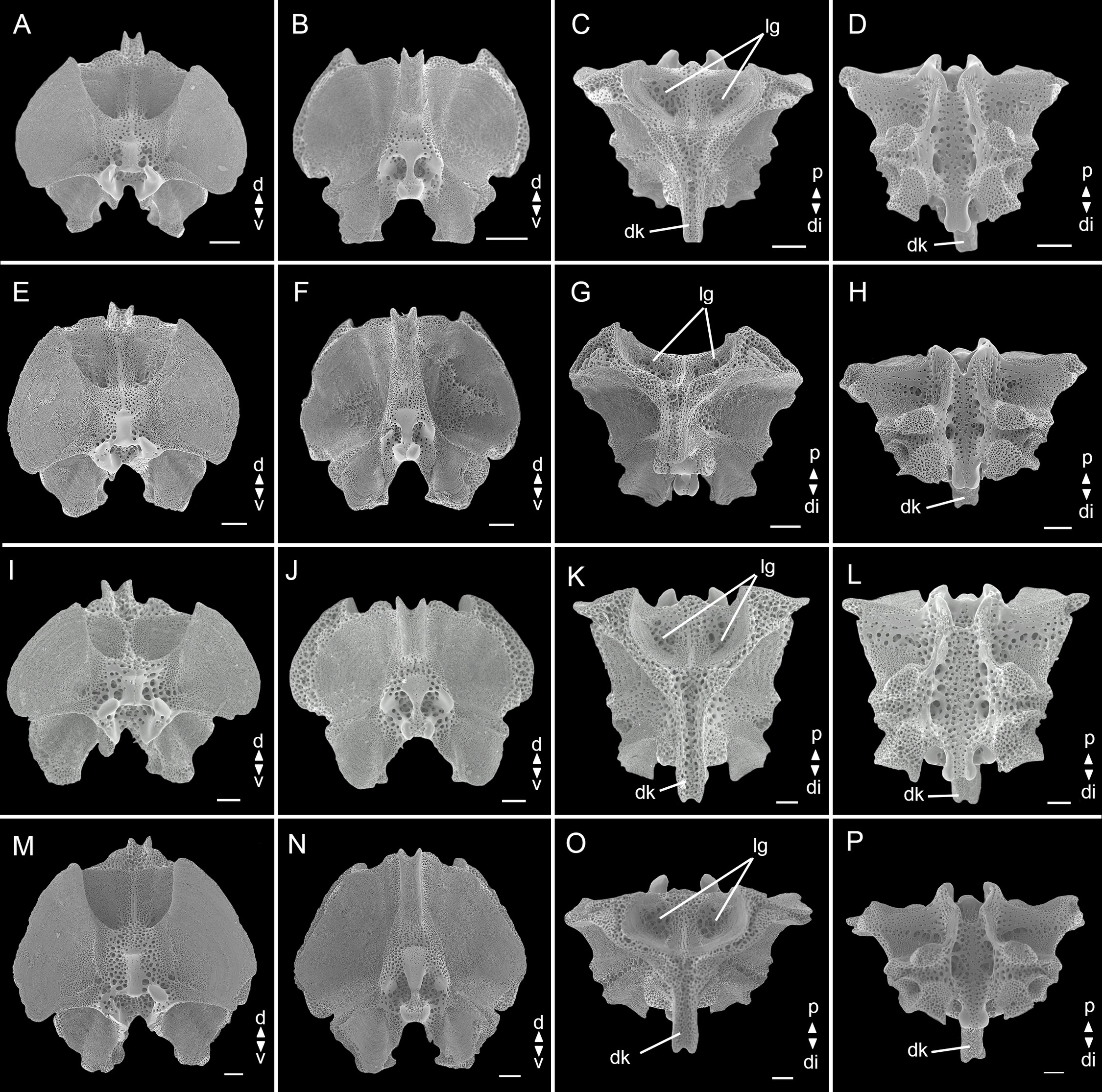

Supplement: S4 Fig — Proximal view (pv), distal view (div), dorsal view (dov), ventral view (vv). CS1 MZUSP 1426: (A) pv; (B) div; (C) dov; (D) vv. CS2 MZUSP 1425: (E) pv; (F) div; (G) dov; (H) vv. CS3 ZUEC OPH 2811: (I) pv; (J) div; (K) dov; (L) vv. CS4 ZUEC OPH 2783: (M) pv; (N) div; (O) dov; (P) vv. d: dorsal; di: distal; dk: distal keel; lg: large groove; p: proximal; v: ventral. Scale bars: 100 μm. (TIF) [file pone.0210331.s004.tif]

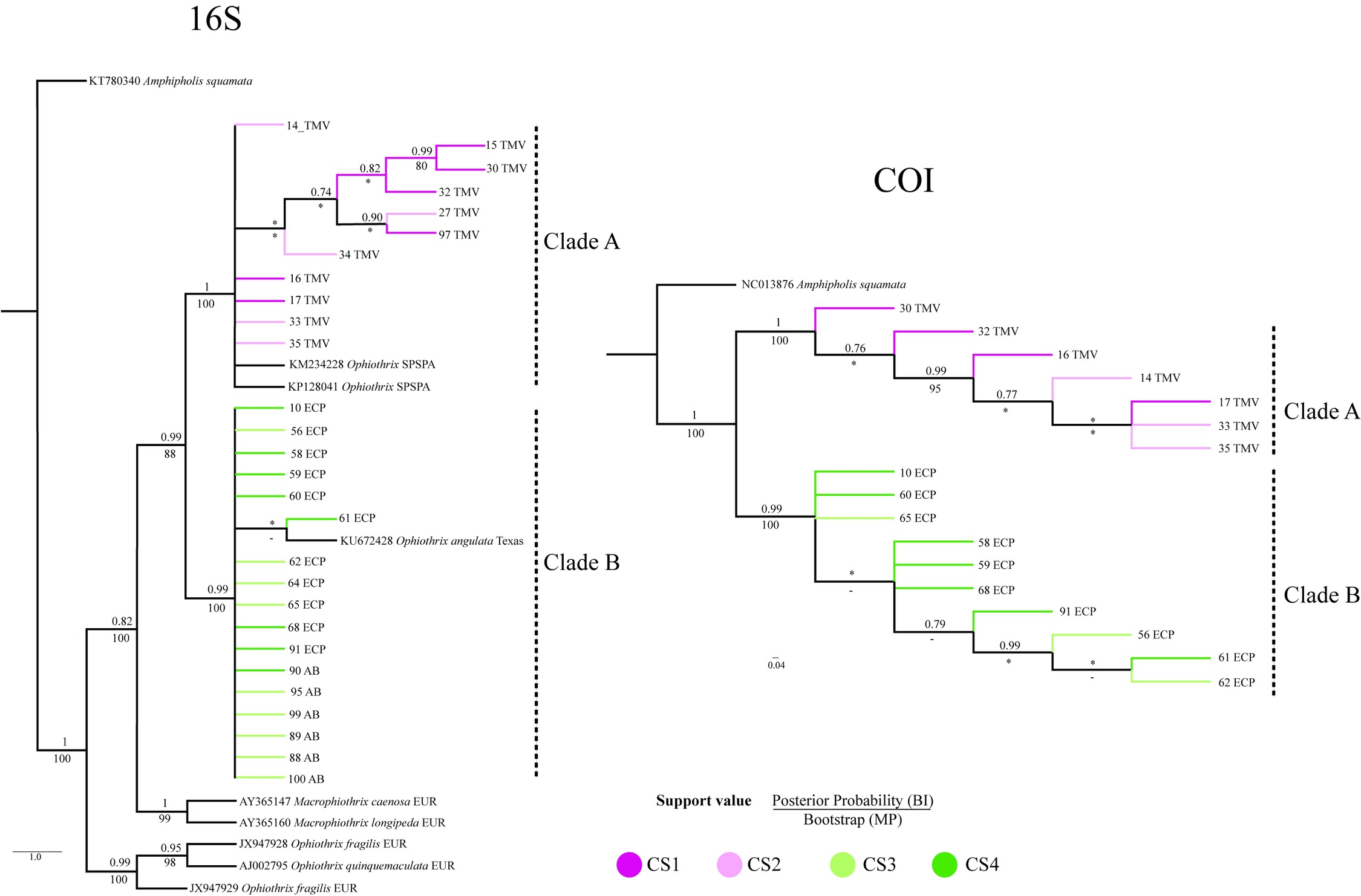

Supplement: S5 Fig — Numbers above and below branches represent the support values for BI (posterior probabilities) and MP (bootstrap values in %), respectively. The MP tree was not represented. Branches are identified by individual codes and their localities (AB: Araçá Bay, São Paulo, Brazil; ECP: Estuarine Complex of Paranaguá, Paraná, Brazil; EUR: Europe; SPSPA: São Pedro and São Paulo Archipelago, Brazil; TMV: Trindade and Martin Vaz Oceanic Archipelago). The scale bar represents the average nucleotide substitutions per site. Asterisks (*) indicates that the support value was lower than 70% (MP) or 0.7 (BI), and a dash (-) indicates that the branch was not recovered. (TIF) [file pone.0210331.s005.tif]

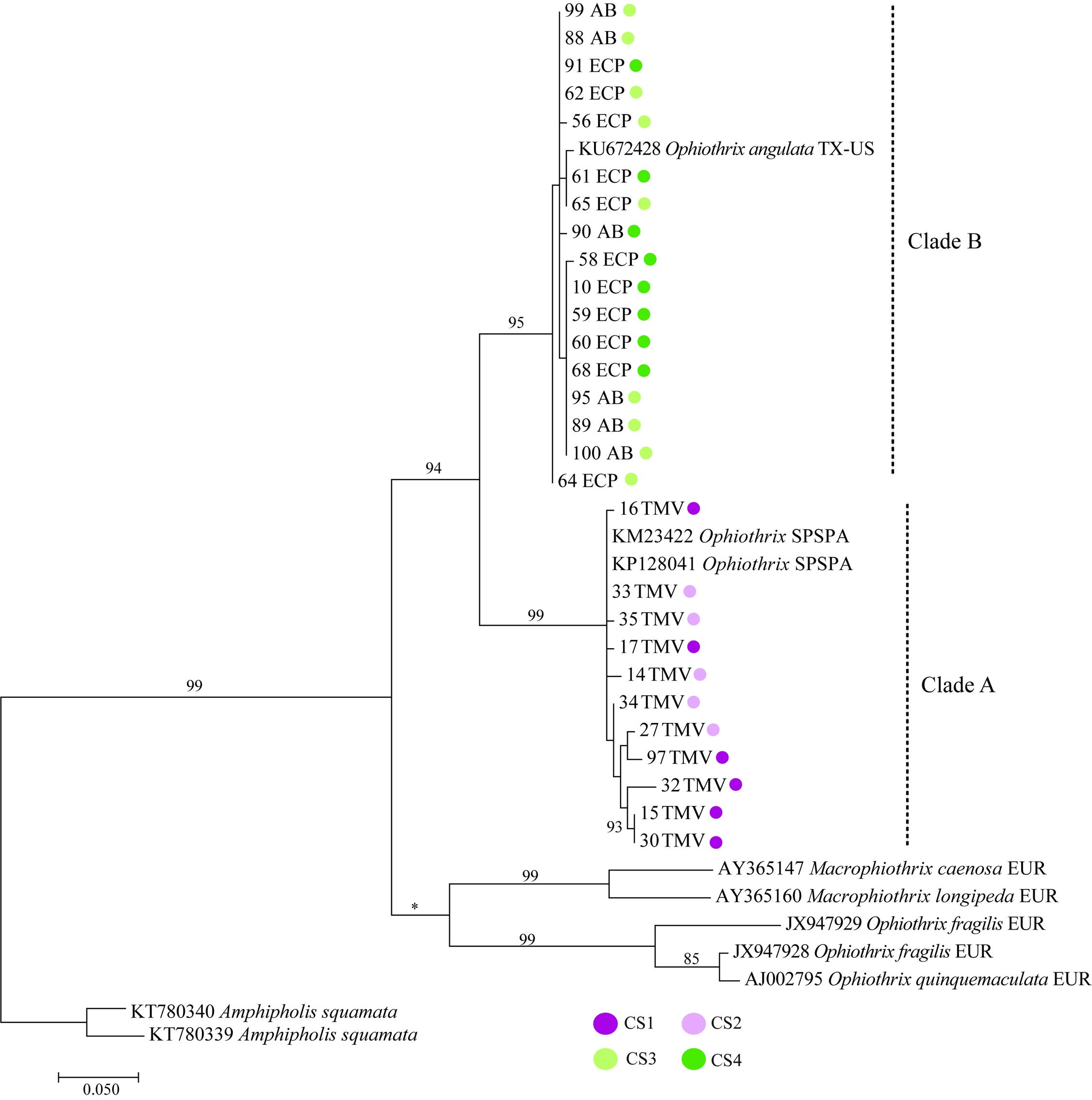

Supplement: S6 Fig — The numbers in nodes represent the support values (bootstrap). Branches are identified by individual codes (S3 Table) and their localities: AB: Araçá Bay, São Paulo, Brazil; ECP: Estuarine Complex of Paranaguá, Paraná, Brazil; EUR: Europe; SPSPA: São Pedro and São Paulo Archipelago, Brazil; TMV: Trindade and Martin Vaz Oceanic Archipelago; TX-US: Texas, United States. The scale bar represents the average nucleotide substitutions per site. Asterisks (*) indicate that the support value was lower than 70%. (TIF) [file pone.0210331.s006.tif]

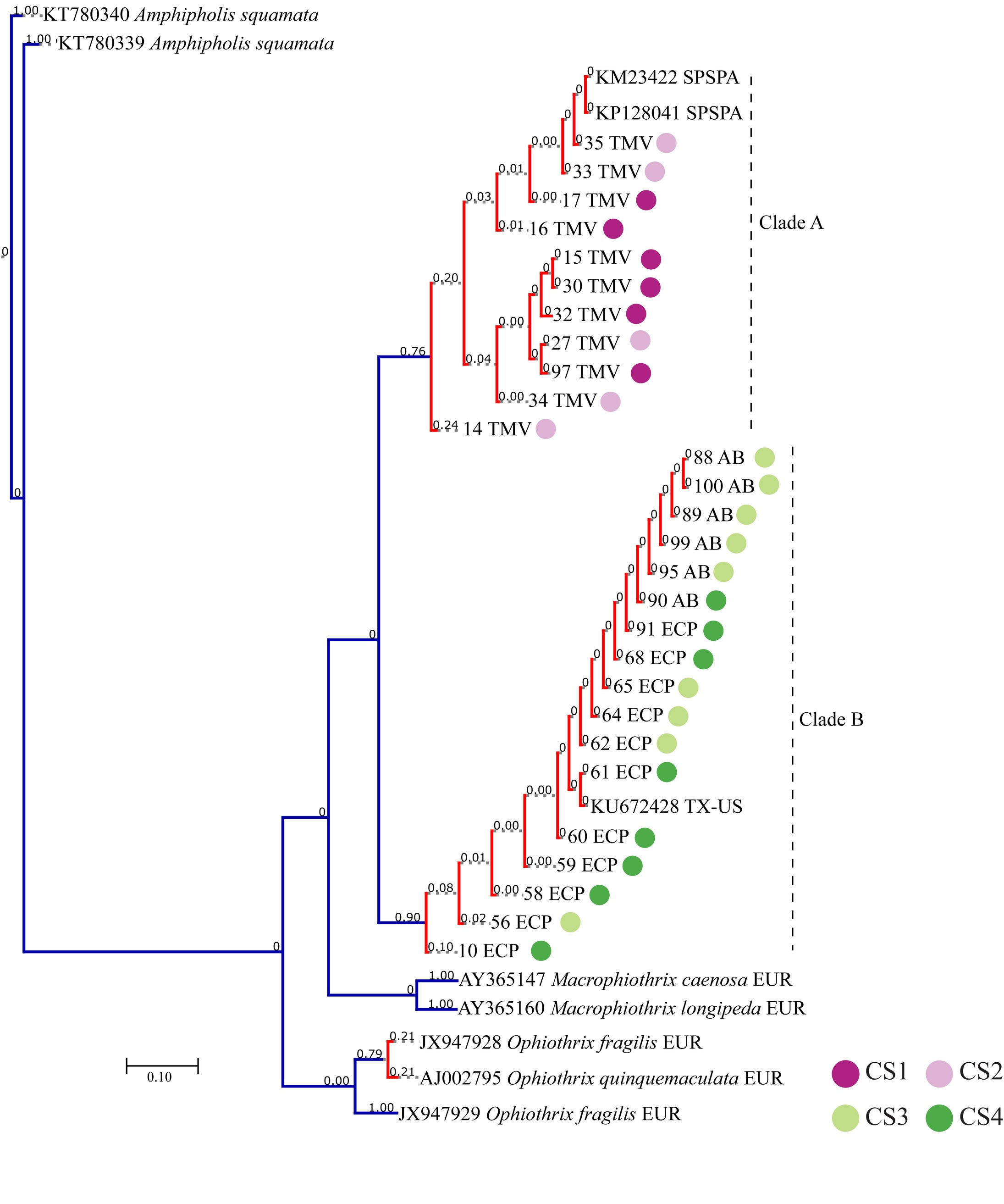

Supplement: S7 Fig — Branches in red connect haplotypes included in same species, in blue distinct species. Branches are identified by individual codes (S3 Table) and their localities: AB: Araçá Bay, São Paulo, Brazil; ECP: Estuarine Complex of Paranaguá, Paraná, Brazil; EUR: Europe; SPSPA: Saint Peter and Saint Paul Archipelago, Brazil; TMV: Trindade and Martin Vaz Oceanic Archipelago; TX-US: Texas, United States. (TIF) [file pone.0210331.s007.tif]

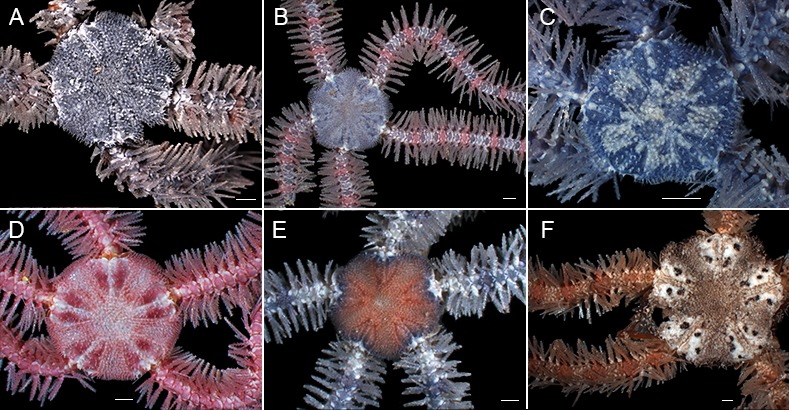

Supplement: S8 Fig — (A) ZUEC OPH 2907, dd = 5.8 mm. (B) MZUSP 1661, dd = 5.3 mm. (C) MZUSP 1687, dd = 3,1 mm. (D) MZUSP 1450, dd = 6.1 mm. (E) MZUSP 1686 dd = 4.2 mm. (F) ZUEC OPH 2883, dd = 8.5 mm. Stereomicroscope photos, scale bar equal to 1 mm. (TIF) [file pone.0210331.s008.tif]
