# Supplementary material for: Atlantic West Ophiothrix spp. in the scope of integrative taxonomy: Confirming the existence of Ophiothrix trindadensis Tommasi, 1970
Source: PLoS One. 2019 Jan 23;14(1):e0210331. doi: 10.1371/journal.pone.0210331 (PMC6343879; doi:10.1371/journal.pone.0210331)
Supplement: S1 Table — AB, Araçá Bay; ECP, Estuarine Complex of Paranaguá; SC-US, South Carolina, United States; SPSPA, São Pedro and São Paulo Islands; TMV, Trindade and Martin Vaz Oceanic Archipelago; TX-US, Texas, United States; USNM, United States National Museum;–absent. (DOCX) [file pone.0210331.s009.docx]

**Table S1.** **Study sites that brittle stars were collected (TMV, AB, ECP) and compared (SPSPA, TX-US, SC-US).**

| Marine Ecoregions | Trindade and Martin Vaz Islands | Southeastern Brazil | | São Pedro and São Paulo Islands | Northern Gulf of Mexico | Carolinian |
| --- | --- | --- | --- | --- | --- | --- |
| Geographic location | TMV | AB | ECP | SPSPA | TX-US | SC-US |
| Project | ProTrindade/CNPq | BIOTA/FAPESP – Araçá | – | PROARQUIPÉLAGO Program | – | From USNM |
| Environment type | Subtidal (7 – 25 m) | Intertidal and subtidal (0 – 20 m) | Intertidal | Subtidal (5 m) | Intertidal | Subtidal (13 m – 66 m) |
| Sampling strategies | Scuba diving | Dredge and by hand | By hand | Scuba diving | By hand | Not informed, but probably by dredge. |
| Geographic coordinates | -20.500, -29.333 (Trindade Island)  -20.500, -28.850 (Martin Vaz Islands) | -23.814, -45.404 | -25.422, -48.407 | 0.917, -29.345 | 27.843, -97.061 | 32.486, -80.353 (Edisto River)  32.097, -80.837 (Calibogue Sound) |
| Substrate type | Rubble bottom and biological substrate (algae) | Rubble bottom and biological substrate (sponge) | Biological substrate (sponge) | Biological substrate (polychaete tubes) | Biological substrate (sandy lobed tunicate colonies) | Not informed. |
| Salinity | ~36 ppt (present study) | ~34 ppt (present study) | ~29 ppt (present study) | ~35 ppt (Becker 2002) | ~30 ppt | Not informed. |
| Water temperature | 25 ºC (present study) | 21 ºC – 24 ºC (present study) | 24 ºC (present study) | 20 ºC – 26 ºC (Becker 2002) | 25 ºC | Not informed |
| References | Almeida [1, 2]; Anker *et al.* [3] | Amaral *et al.* [4,5]; Siegle *et al.* [6] | Lessa *et al.* [7], Lana *et al.* [8] | Edwards & Lubbock [9,10]; Barboza *et al.* [11]; Viana *et al.* [12] | The University of Texas at Austin [13,14] | – |

AB, Araçá Bay; ECP, Estuarine Complex of Paranaguá; SC-US, South Carolina, United States; SPSPA, São Pedro and São Paulo Islands; TMV, Trindade and Martin Vaz Oceanic Archipelago; TX-US, Texas, United States; USNM, United States National Museum; – absent.

References:

1. Almeida FFM. Ilha de Trindade – registro de vulcanismo cenozóico no Atlântico Sul. In: Schobbenhaus C, Campos DA, Queiroz ET, Winge M, Berbert-Born MLC, editors. Sítios geológicos e paleontológicos do Brasil. Brasília: DNPM/CPRM, Comissão Brasileira de Sítios Geológicos e Paleobiológicos (SIGEP); 2002. pp. 369–377.
2. Almeida FFM. Ilhas oceânicas brasileiras e suas relações com a tectônica atlântica. Terrae Didat. 2006;2: 3–18.
3. Anker A, Tavares M, Mendonça JB. Alpheid shrimps (Decapoda: Caridea) of the Trindade & Martin Vaz Archipelago, off Brazil, with new records, description of a new species of Synalpheus and remarks on zoogeographical patterns in the oceanic islands of the tropical southern Atlantic. Zootaxa. 2016;4138: 1–58. doi: 10.11646/zootaxa.4138.1.1
4. Amaral ACZ, Migotto AE, Turra A, Schaeffer-Novelli Y. Araçá: biodiversidade, impactos e ameaças. Biota Neotrop. 2010;10: 219–264. doi: 10.1590/s1676-06032010000100022
5. Amaral ACZ, Turra A, Ciotti AM, Rossi-Wongstschowski CLDB, Schaeffer-Novelli Y. Life in Araçá Bay: diversity and importance. 3rd ed. São Paulo: Lume; 2016. <http://www.bibliotecadigital.unicamp.br/document/?code=73819&opt=1>
6. Siegle E, Dottori M, Villamarin BC. Hydrodynamics of a subtropical tidal flat: Araçá Bay, Brazil. Ocean & Coastal Management. 2017;in press: doi: 10.1016/j.ocecoaman.2017.11.003
7. Lessa GC, Meyers SR, Marone E. Holocene stratigraphy in the Paranagua Bay estuary, southern Brazil. Journal of Sedimentary Research. 1998;68: 1060–1076.
8. Lana PC, Marone E, Lopes RM, Machado EC. The Subtropical Estuarine Complex of Paranaguá Bay, Brazil. In: Seeliger U, Kjerfve B, editors. Coastal Marine Ecosystems of Latin America. Berlin, Heidelberg: Springer Berlin Heidelberg; 2001. pp. 131–145.
9. Edwards A, Lubbock R. Marine Zoogeography of St Paul's Rocks. Journal of Biogeography. 1983a;10: 65–72. doi: 10.2307/2844583
10. Edwards A, Lubbock R. The ecology of Saint Paul's Rocks (Equatorial Atlantic). J Zool. 1983b;200: 51–69. doi: 10.1111/j.1469-7998.1983.tb06108.x
11. Barboza CAM, Mattos G, Paiva PC. Brittle stars from the Saint Peter and Saint Paul Archipelago: morphological and molecular data. Mar Biodivers Rec. 2015;8: 1–9. doi: 10.1017/S1755267214001511
12. Viana DL, Hazin FHV, Oliveira JEL, Souza MAC. Saint Peter and Saint Paul archipelago: Brazil in the mid atlantic. Recife: Vedas Edições; 2017.
13. The University of Texas at Austin. The Texas High School Coastal Monitoring Program 2006. Available from: <http://www.beg.utexas.edu/coastal/thscmp/fg_mustang_6.htm>.
14. The University of Texas at Austin. Marine Science Institute College of Natural Sciences 2018. Available from: <https://utmsi.utexas.edu/>.
