# Supplementary material for: Atlantic West Ophiothrix spp. in the scope of integrative taxonomy: Confirming the existence of Ophiothrix trindadensis Tommasi, 1970
Source: PLoS One. 2019 Jan 23;14(1):e0210331. doi: 10.1371/journal.pone.0210331 (PMC6343879; doi:10.1371/journal.pone.0210331)
Supplement: S2 Table — (DOCX) [file pone.0210331.s010.docx]

**Table S2.** **Abbreviations and definitions of the 17 morphological characters used for the morphometric analysis.**

| Abbreviation | Character name | Definition |
| --- | --- | --- |
| dd | Disc diameter | Length from the distal edge of the radial shield to the interradial edge of the disc (Fig. S2 B) |
| rs_l | Radial shield length | Length of one radial shield (Fig. S2 A) |
| rs_w | Radial shield width | Width of one radial shield (Fig. S2 A) |
| disc_spine | Disc spine | Length of the largest denticulate spine (as those of the arms) near to the disc center |
| as | Arm spine | Number of arm spines at the third free arm segment (Fig. S2 B) |
| as2 | Second arm spine | Length of the second arm spine at the third free arm segment (Fig. S2 B) |
| od | Oral diameter | Length from the proximal edge of adoral shield to distal edge of the first ventral arm plate (Fig. S2 E) |
| os_l | Oral shield length | Length of one oral shield (Fig. S2 D) |
| os_w | Oral shield width | Width of one oral shield (Fig. S2 D) |
| ads_l | Adoral shield length | Length of one adoral shield (Fig. S2 D) |
| ads_w | Adoral shield width | Width of one adoral shield (Fig. S2 D) |
| dap_l | Dorsal arm plate length | Length of the third dorsal arm plate at the free arm segment (Fig. S2 C) |
| dap_w | Dorsal arm plate width | Width of the third dorsal arm plate at the free arm segment (Fig. S2 C) |
| vap1_l | 1^st^ ventral arm plate length | Length of the first ventral arm plate (Fig. S2 F) |
| vap1_w | 1^st^ Ventral arm plate width | Width of the first ventral arm plate (Fig. S2 F) |
| vap2_l | 2^nd^ ventral arm plate length | Length of the second ventral arm plate (Fig. S2 F) |
| vap2_w | 2^nd^ ventral arm plate width | Width of the second ventral arm plate (Fig. S2 F) |
