# Supplementary material for: Atlantic West Ophiothrix spp. in the scope of integrative taxonomy: Confirming the existence of Ophiothrix trindadensis Tommasi, 1970
Source: PLoS One. 2019 Jan 23;14(1):e0210331. doi: 10.1371/journal.pone.0210331 (PMC6343879; doi:10.1371/journal.pone.0210331)
Supplement: S3 Table — AB, Araçá Bay, Brazil; AM, Australian Museum; ECP, Estuarine Complex of Paranaguá, Brazil; EUR, Europe; FMC, French Mediterranean Coast; IC, individual codes in the present study; NMRJ, National Museum of Rio de Janeiro, Brazil; ROS, Roscoff/France; SPSPA, São Pedro and São Paulo Archipelago; TMV, Trindade and Martin Vaz Oceanic Archipelago, Brazil; TX-US, Texas, United States; ZUEC OPH, Scientific Collection of Ophiuroidea in Museum of Zoology of the University of Campinas. (DOCX) [file pone.0210331.s011.docx]

**Table S3.** **Specimens used in the phylogenetic analyses with their locality, museums vouchers, and GenBank accession numbers for DNA sequences.**

| **Museum voucher** | **IC** | **Species** | **Locality** | **GenBank (16S)** | **GenBank (COI)** | **Reference** |
| --- | --- | --- | --- | --- | --- | --- |
| MZUSP 1425 | 14 | *Ophiothrix trindadensis* | TMV | MH281576 | MH281604 | Neotype – present study |
| MZUSP 1426 | 15 | *Ophiothrix trindadensis* | TMV | MH281577 |  | Present study |
| MZUSP 1427 | 16 | *Ophiothrix trindadensis* | TMV | MH281578 | MH281605 | Present study |
| MZUSP 1428 | 17 | *Ophiothrix trindadensis* | TMV | MH281579 | MH281606 | Present study |
| MZUSP 1429 | 27 | *Ophiothrix trindadensis* | TMV | MH281580 |  | Present study |
| MZUSP 1430 | 30 | *Ophiothrix trindadensis* | TMV | MH281581 | MH281607 | Present study |
| ZUEC OPH 2813 | 32 | *Ophiothrix trindadensis* | TMV | MH281582 | MH281608 | Present study |
| ZUEC OPH 2880 | 33 | *Ophiothrix trindadensis* | TMV | MH281583 | MH281609 | Present study |
| ZUEC OPH 2881 | 34 | *Ophiothrix trindadensis* | TMV | MH281584 |  | Present study |
| ZUEC OPH 2882 | 35 | *Ophiothrix trindadensis* | TMV | MH281585 | MH281610 | Present study |
| ZUEC OPH 2883 | 97 | *Ophiothrix trindadensis* | TMV | MH281586 |  | Present study |
| ZUEC OPH 2803 | 10 | *Ophiothrix* cf. *angulata* | ECP | MH281587 | MH281613 | Present study |
| ZUEC OPH 2149 | 100 | *Ophiothrix angulata* | AB | MH281603 |  | Present study |
| ZUEC OPH 2802 | 99 | *Ophiothrix angulata* | AB | MH281600 |  | Present study |
| ZUEC OPH 2811 | 95 | *Ophiothrix angulata* | AB | MH281599 |  | Present study |
| ZUEC OPH 2785 | 91 | *Ophiothrix* cf. *angulata* | ECP | MH281597 | MH281620 | Present study |
| ZUEC OPH 2455 | 90 | *Ophiothrix* cf. *angulata* | AB | MH281598 |  | Present study |
| ZUEC OPH 2796 | 89 | *Ophiothrix angulata* | AB | MH281601 |  | Present study |
| MZUSP 1696 | 88 | *Ophiothrix angulata* | AB | MH281602 |  | Present study |
| ZUEC OPH 2783 | 68 | *Ophiothrix* cf. *angulata* | ECP | MH281596 | MH281619 | Present study |
| ZUEC OPH 2808 | 65 | *Ophiothrix angulata* | ECP | MH281595 | MH281618 | Present study |
| ZUEC OPH 2807 | 64 | *Ophiothrix angulata* | ECP | MH281594 |  | Present study |
| MZUSP 1695 | 62 | *Ophiothrix angulata* | ECP | MH281593 | MH281617 | Present study |
| ZUEC OPH 2799 | 61 | *Ophiothrix* cf. *angulata* | ECP | MH281592 | MH281616 | Present study |
| ZUEC OPH 2798 | 60 | *Ophiothrix* cf. *angulata* | ECP | MH281591 | MH281615 | Present study |
| ZUEC OPH 2797 | 59 | *Ophiothrix* cf. *angulata* | ECP | MH281590 | MH281614 | Present study |
| ZUEC OPH 2792 | 58 | *Ophiothrix* cf. *angulata* | ECP | MH281589 | MH281612 | Present study |
| ZUEC OPH 2805 | 56 | *Ophiothrix angulata* | ECP | MH281588 | MH281611 | Present study |
| NMRJ |  | *Ophiothrix angulata* | SPSPA | KM234228 |  | 1 |
| NMRJ |  | *Ophiothrix angulata* | SPSPA | KP128041 |  | 1 |
| Absent |  | *Ophiothrix angulata* | TX-US | KU672428 |  | 2 |
| Absent |  | *Ophiothrix fragilis* | EUR | JX947928 |  | 3 |
| Absent |  | *Ophiothrix fragilis* | EUR | JX947929 |  | 3 |
| Absent |  | *Ophiothrix quinquemaculata* | EUR | AJ002795 |  | 4 |
| AM |  | *Macrophiothrix caenosa* | EUR | AY365147 |  | 5 |
| AM |  | *Macrophiothrix longipeda* | EUR | AY365160 |  | 5 |
| Absent |  | *Amphipholis squamata* | FMC | KT780340 |  | 6 |
| Absent |  | *Amphipholis squamata* | FMC | KT780339 |  | 6 |
| Absent |  | *Amphipholis squamata* | ROS |  | NC013876 | 7 |

AB, Araçá Bay, Brazil; AM, Australian Museum; ECP, Estuarine Complex of Paranaguá, Brazil; EUR, Europe; FMC, French Mediterranean Coast; IC, individual codes in the present study; MZUSP, Museum of Zoology of the University of São Paulo; NMRJ, National Museum of Rio de Janeiro, Brazil; ROS, Roscoff/France; SPSPA, São Pedro and São Paulo Archipelago; TMV, Trindade and Martin Vaz Oceanic Archipelago, Brazil; TX-US, Texas, United States; ZUEC OPH, Scientific Collection of Ophiuroidea in Museum of Zoology of the University of Campinas.

References

1. Barboza CAM, Mattos G, Paiva PC. Brittle stars from the Saint Peter and Saint Paul Archipelago: morphological and molecular data. Mar Biodivers Rec. 2015;8: 1–9. doi: 10.1017/S1755267214001511
2. Hunter RL, Brown LM, Alexander Hill C, Kroeger ZA, Rose SE. Additional insights into phylogenetic relationships of the Class Ophiuroidea (Echinodermata) from rRNA gene sequences. Journal of Zoological Systematics and Evolutionary Research. 2016;1–7. doi: 10.1111/jzs.12135
3. Pérez‐Portela R, Almada V, Turon X. Cryptic speciation and genetic structure of widely distributed brittle stars (Ophiuroidea) in Europe. Zool Scripta. 2013;42: 151–169. doi: 10.1111/j.1463-6409.2012.00573.x
4. Baric S, Sturmbauer C. Ecological Parallelism and Cryptic Species in the Genus *Ophiothrix* Derived from Mitochondrial DNA Sequences. Mol Phylogenet Evol. 1999;11: 157­–162. doi: 10.1006/mpev.1998.0551
5. Hart MW, Podolsky RD. Mitochondrial DNA phylogeny and rates of larval evolution in *Macrophiothrix* *brittlestars*. Mol Phylogenet Evol. 2005;34: 438–447. doi: 10.1016/j.ympev.2004.09.011
6. Boissin E, Egea E, Féral J, Chenuil A. Contrasting population genetic structures in *Amphipholis* *squamata*, a complex of brooding, self-reproducing sister species sharing life history traits. Marine Ecology Progress Series. 2015;539: 165–177. doi: 10.3354/meps11480
7. Perseke M, Bernhard D, Fritzsch G, Brümmer F, Stadler PF, Schlegel M. Mitochondrial genome evolution in Ophiuroidea, Echinoidea, and Holothuroidea: insights in phylogenetic relationships of Echinodermata. Mol Phylogenet Evol. 2010;56: 201–211. doi: 10.1016/j.ympev.2010.01.035
