# Supplementary material for: Atlantic West Ophiothrix spp. in the scope of integrative taxonomy: Confirming the existence of Ophiothrix trindadensis Tommasi, 1970
Source: PLoS One. 2019 Jan 23;14(1):e0210331. doi: 10.1371/journal.pone.0210331 (PMC6343879; doi:10.1371/journal.pone.0210331)
Supplement: S4 Table — (DOCX) [file pone.0210331.s012.docx]

**Table S4.** **Comparative table of the differences in dorsal and lateral arm plates between the CS.**

| Character | CS1, CS2 | CS3, CS4 |
| --- | --- | --- |
| Dorsal arm plates | One and a half times as wide as long  Distal region three to four times greater than the proximal one  With a prominent keel at median portion | As wide as long  Distal and proximal regions at the same width  Without a prominent keel at median portion |
| Lateral arm plates | Eight or more spine articulations | At most seven spine articulations |
