# Supplementary material for: Atlantic West Ophiothrix spp. in the scope of integrative taxonomy: Confirming the existence of Ophiothrix trindadensis Tommasi, 1970
Source: PLoS One. 2019 Jan 23;14(1):e0210331. doi: 10.1371/journal.pone.0210331 (PMC6343879; doi:10.1371/journal.pone.0210331)
Supplement: S5 Table — Genetic distance between the CSs estimated from the mitochondrial gene fragments 16S (downward triangle) and COI (top triangle). In the gray diagonal line, average genetic distance (%) of both genes within the groups of sequences. AB, Araçá Bay, São Paulo, Brazil; CS, Candidate Species inferred from morphological characters; ECP, Estuarine Complex of Paranaguá, Paraná, Brazil; EUR, Europe; Nc, Value not calculated because of the inexistence of groups’ sequences for the considered gene; SPSPA, Saint Peter and Saint Paul Archipelago, Brazil; TMV, Trindade and Martin Vaz Oceanic Archipelago; TX-US, Texas, United States. # value of genetic distance within the group not calculated because only one sequence was considered in the analysis. (DOCX) [file pone.0210331.s013.docx]

**Table S5.** **Genetic distance (%) between the CS.**

|  | Taxa groups | 1 | 2 | 3 | 4 | 5 | 6 | 7 | 8 | 9 |
| --- | --- | --- | --- | --- | --- | --- | --- | --- | --- | --- |
| 1 | *Ophiothrix* CS1 – TMV | 1.6 | 3.5 | 17.0 | 16.8 | Nc | Nc | Nc | Nc | 24.9 |
| 2 | *Ophiothrix* CS2 – TMV | 1.4 | 0.9 | 17.2 | 17.0 | Nc | Nc | Nc | Nc | 25.1 |
| 3 | *Ophiothrix* CS3 – ECP, AB | 12.0 | 11.6 | 0.4 | 1.5 | Nc | Nc | Nc | Nc | 24.7 |
| 4 | *Ophiothrix* CS4 – ECP, AB | 11.9 | 11.4 | 0.4 | 0.4 | Nc | Nc | Nc | Nc | 24.8 |
| 5 | *Ophiothrix angulata* SPSPA | 1.2 | 0.6 | 11.3 | 11.2 | 0 | Nc | Nc | Nc | Nc |
| 6 | *Ophiothrix angulata* TX-US | 12.1 | 11.7 | 0.8 | 0.9 | 11.4 | # | Nc | Nc | Nc |
| 7 | *Ophiothrix* spp. EUR | 23.2 | 22.8 | 20.5 | 20.8 | 22.9 | 20.3 | 7.3 | Nc | Nc |
| 8 | *Macrophiothrix* spp. EUR | 19.2 | 19.1 | 19.3 | 19.2 | 18.8 | 18.9 | 22.6 | 9.9 | Nc |
| 9 | *Amphipholis squamata* | 31.2 | 31.1 | 32.6 | 32.4 | 30.4 | 32.5 | 35.0 | 34.7 | 4.6 |

Genetic distance between the CSs estimated from the mitochondrial gene fragments 16S (downward triangle) and COI (top triangle). In the gray diagonal line, average genetic distance (%) of both genes within the groups of sequences.

AB, Araçá Bay, São Paulo, Brazil; CS, Candidate Species inferred from morphological characters; ECP, Estuarine Complex of Paranaguá, Paraná, Brazil; EUR, Europe; Nc, Value not calculated because of the inexistence of groups’ sequences for the considered gene; SPSPA, Saint Peter and Saint Paul Archipelago, Brazil; TMV, Trindade and Martin Vaz Oceanic Archipelago; TX-US, Texas, United States. # value of genetic distance within the group not calculated because only one sequence was considered in the analysis.
