# Supplementary material for: Atlantic West Ophiothrix spp. in the scope of integrative taxonomy: Confirming the existence of Ophiothrix trindadensis Tommasi, 1970
Source: PLoS One. 2019 Jan 23;14(1):e0210331. doi: 10.1371/journal.pone.0210331 (PMC6343879; doi:10.1371/journal.pone.0210331)
Supplement: S6 Table — Group I is composed of the specimens from Trindade and Martin Vaz Oceanic Archipelago, Saint Peter and Saint Paul Archipelago–which corresponds to Clade A inferred in the phylogenetic analysis. Group II is composed of the specimens from Estuarine Complex of Paranaguá, Araçá Bay, and Texas–which corresponds to Clade B inferred in the phylogenetic analysis. (DOCX) [file pone.0210331.s014.docx]

**Table S5.** **Analysis of Molecular Variance (AMOVA) for the population structuring scenario given by the groups I and II.**

| **Source of variation** | **d.f.** | **Sum of squares** | **Variance components** | **Percentage of variation** |
| --- | --- | --- | --- | --- |
| Among groups | 1 | 268.700 | 17.72975 Va | 93.49 |
| Among populations within groups | 3 | 3.474 | -0.02370 Vb | -0.12 |
| Within populations | 26 | 32.697 | 1.25758 Vc | 6.63 |
| Total | 30 | 304.871 | 18.96363 |  |

Group I is composed of the specimens from Trindade and Martin Vaz Oceanic Archipelago, Saint Peter and Saint Paul Archipelago – which corresponds to Clade A inferred in the phylogenetic analysis. Group II is composed of the specimens from Estuarine Complex of Paranaguá, Araçá Bay, and Texas – which corresponds to Clade B inferred in the phylogenetic analysis.
